# The Structure of Genetic Diversity in Eelgrass (*Zostera marina* L.) along the North Pacific and Bering Sea Coasts of Alaska

**DOI:** 10.1371/journal.pone.0152701

**Published:** 2016-04-22

**Authors:** Sandra L. Talbot, George K Sage, Jolene R. Rearick, Meg C. Fowler, Raquel Muñiz-Salazar, Bethany Baibak, Sandy Wyllie-Echeverria, Alejandro Cabello-Pasini, David H. Ward

**Affiliations:** 1 Alaska Science Center, U.S. Geological Survey, Anchorage, Alaska, United States of America; 2 Department of Biology, University of New Mexico, Albuquerque, New Mexico, United States of America; 3 Escuela de Ciencias de la Salud, Universidad Autónoma de Baja California, Ensenada, Baja California, Mexico; 4 Biological Sciences, Humboldt State University, Arcata, California, United States of America; 5 Friday Harbor Laboratories, University of Washington, Friday Harbor, Washington, United States of America; 6 Center for Marine and Environmental Studies, University of Virgin Islands, St. Thomas, Virgin Islands, United States of America; 7 Instituto de Investigaciones Oceanológicas, Universidad Autónoma de Baja California, California, Mexico; Chinese Academy of Sciences, CHINA

## Abstract

Eelgrass (*Zostera marina)* populations occupying coastal waters of Alaska are separated by a peninsula and island archipelago into two Large Marine Ecosystems (LMEs). From populations in both LMEs, we characterize genetic diversity, population structure, and polarity in gene flow using nuclear microsatellite fragment and chloroplast and nuclear sequence data. An inverse relationship between genetic diversity and latitude was observed (heterozygosity: R^2^ = 0.738, *P <* 0.001; allelic richness: R^2^ = 0.327, P = 0.047), as was significant genetic partitioning across most sampling sites (θ = 0.302, *P <* 0.0001). Variance in allele frequency was significantly partitioned by region only in cases when a population geographically in the Gulf of Alaska LME (Kinzarof Lagoon) was instead included with populations in the Eastern Bering Sea LME (θ_p_ = 0.128–0.172; *P <* 0.003), suggesting gene flow between the two LMEs in this region. Gene flow among locales was rarely symmetrical, with notable exceptions generally following net coastal ocean current direction. Genetic data failed to support recent proposals that multiple *Zostera* species (i.e. *Z*. *japonica* and *Z*. *angustifolia*) are codistributed with *Z*. *marina* in Alaska. Comparative analyses also failed to support the hypothesis that eelgrass populations in the North Atlantic derived from eelgrass retained in northeastern Pacific Last Glacial Maximum refugia. These data suggest northeastern Pacific populations are derived from populations expanding northward from temperate populations following climate amelioration at the terminus of the last Pleistocene glaciation.

## Introduction

Eelgrass (*Zostera marina* Linnaeus, SP.PL.2: 968. 1753), a marine angiosperm adapted to the cold waters of the North Atlantic and North Pacific, is the most widely distributed of the 60+ seagrass species. *Zostera marina* provides valuable habitat for diverse animal assemblages, functioning as an important primary producer and erosion stabilizer in coastal ecosystems. Dramatic declines in seagrasses are documented worldwide over the past three decades [1–3]. Major declines of eelgrass populations in North America have been attributed to a variety of both human-induced events, such as the release of oil, farming induced eutrophication, and residential expansion [4,5], and natural events, such as disease and anoxia [6–8]. Although the most severe declines in eelgrass populations in North America have occurred along the Atlantic coast, populations on the Pacific coast have also experienced losses, especially in the southern portion of the range [1,9,10].

Coastal Alaska, on the Pacific coast of North America, represents both the northern- and western-most limits of *Z*. *marina* in the Northeast Pacific region [11], and eelgrass meadows there likely comprise the largest contiguous seagrass meadows in northern North America [12,13]. Despite a recent proposal that another species, *Z*. *angustifolia*, occurs in mixed stands with *Z*. *marina* in some Alaskan locales [14], and that the *Z*. *japonica*, an invasive, nonindigenous species [15] occurs in southeastern Alaskan waters (i.e. UAM Herb: 248738–41), prior genetic analyses based on sequence data from nuclear and chloroplast genes in a small number of eelgrass specimens collected from six locales in Alaska [16] suggests that only one species of *Zostera* (*Z*. *marina*) occurs in Alaskan waters [17]. Along the coast of Alaska, *Z*. *marina* occurs in geographically isolated coastal lagoons in two Large Marine Ecosystems (LMEs) [18], the Eastern Bering Sea and Gulf of Alaska LMEs (hereafter, EBS-LME and GoA-LME, respectively), and is thought to be the dominant submerged macrophyte in both ecosystems [12,13,19]. Due in part to the lack of heavy anthropogenic activity along remote areas of the Alaskan coastline (particularly in the western portion), eelgrass populations here have not suffered declines as found elsewhere along the Pacific coast of North America [9,10] (e.g., Baja California [9,10], California, Oregon and Washington [1]), in the Northwest Pacific region (e.g., Japan [20,21]), and in more densely populated regions including the Atlantic coast of North America and Europe [22]. However, natural processes (e.g., seismic activity [23]) will continue to impact high latitude eelgrass populations, and direct or indirect impacts due to localized human activity (e.g., development of boat harbors [24] and oil spills [25]) as well as large scale climate change [25,26], are expected to increase in high latitude communities in the future.

**Fig 1:**
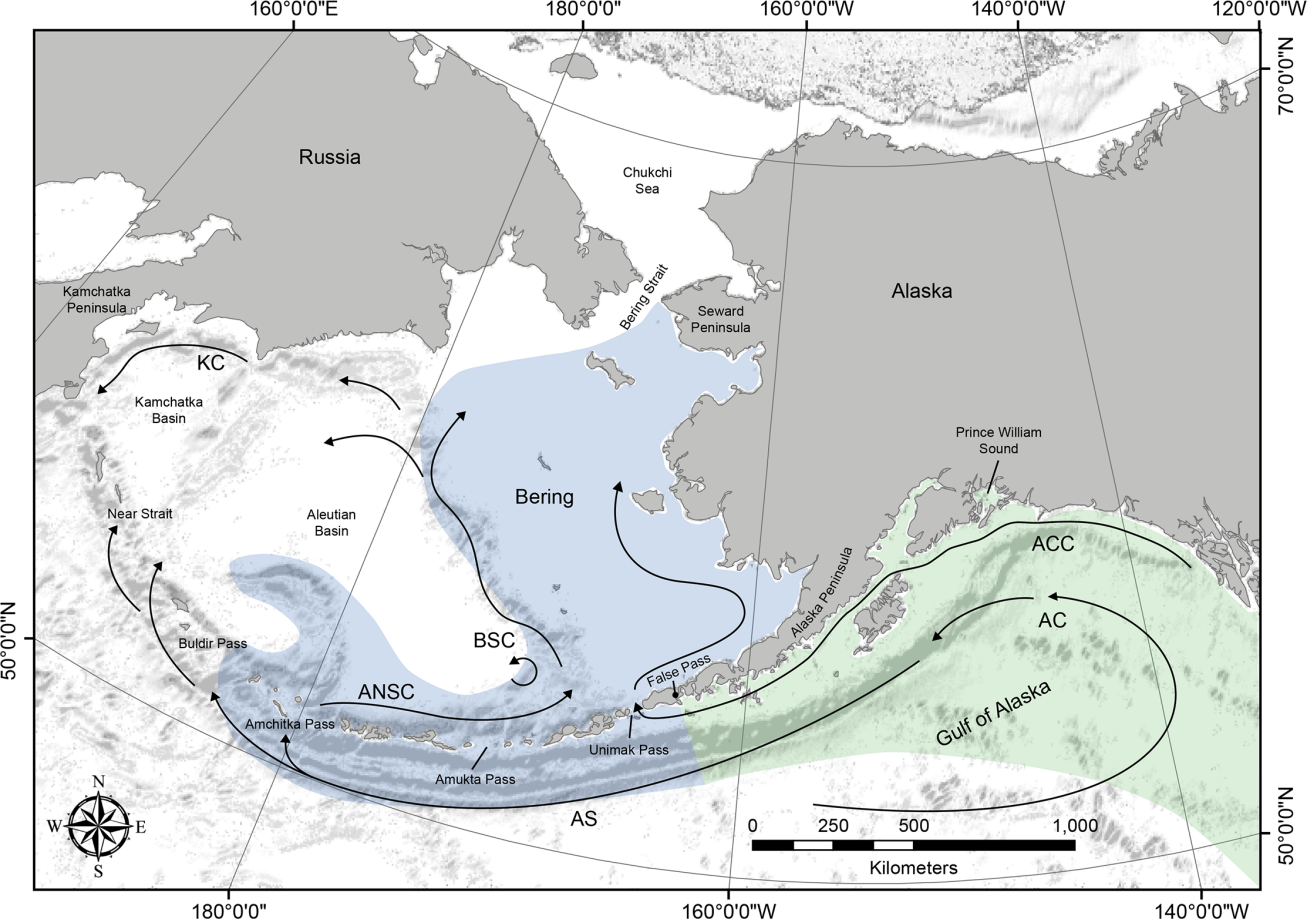
Major prevailing ocean currents and passes in the Aleutian Islands, within the EBS- and GoA-LMEs. The EBS-LME is shown in blue; the GoA-LME is shown in green. Major currents are the Alaska Current (AC), Alaska Coastal Current (ACC), Alaska Stream (AS), Aleutian North Slope Current (ANSC), Bering Sea Current (BSC), and Kamchatka Current (KC). Gray areas in the water indicate trenches. Ocean current data are compiled, but not identical to, figures from Stabeno and Reed [30,31]. The base map was produced using ArcGIS version 10.2.2 [32].

Eelgrass populations along the Alaska Peninsula (Fig 1) and the eastern portion of the Aleutian Archipelago are of considerable phytogeographic interest because they occur along the southern margin of what was once the Bering Land Bridge. The Alaska Peninsula, together with the Aleutian Archipelago, functions as an important intercontinental bridge for dispersal for both marine and terrestrial organisms. For species that occur in coastal marine and terrestrial habitats along the Alaska Peninsula and the Aleutian chain, evolutionary dispersal (gene flow) between continents can occur in at least two directions: westward from North America along the Alaska Peninsula through the Aleutian Island Archipelago to Kamchatka, and eastward from Eurasia, toward interior Alaska and the Pacific coast [27]. However, native eelgrass populations are generally thought to be absent along the Aleutian Archipelago beyond the easternmost (“Fox”) island group [28]; the population on Adak Island, in the Andreanof Island group in the central Aleutian Islands, is considered to be the result of a transplantation experiment conducted in the 1960s [29]. Nevertheless, McRoy [29] suggests Atka Island, also in the Andreanof Island group, hosts eelgrass populations. Thus, while we are unaware of the existence of any specimens of *Z*. *marina* collected from Atka, its presence there could signal a phylogeographic connection with eelgrass populations in Eurasia.

Populations of *Z*. *marina* along the southern (Pacific coast) of the Alaska Peninsula (part of the GoA-LME) are separated geographically from populations along the northern coast of the peninsula (part of the EBS-LME), and exposed to different current regimes and environmental conditions. Differences in gene flow polarity among populations of marine organisms in the different LMEs may reflect the different ocean current circulation patterns. On the Pacific side of the peninsula, the Alaska Stream, flows westward along the southern portion of the Alaska Peninsula and represents the northern boundary current of the anticyclonic Pacific subarctic gyre (Fig 1), which extends from the head of the Gulf of Alaska to the western Aleutians (165° W to 173° E), and then turns northwesterly into the Bering Sea at Amchitka Pass (180° E). Unlike other oceanic currents, which experience wide variations in localized kinetic energy (such as the Gulf Stream), the Alaska Stream is a narrow and consistent high speed current [30], which may impact polarity of gene flow among populations of marine organisms [30,33].

Circulation is predominantly cyclonic (counter clockwise) in the Bering Sea basin [34]; the Aleutian North Slope Current flows eastward along the northern slope of the Aleutian Islands, then turns northwestern to form the Bering Slope Current, whereas the southward flowing Kamchatka Current forms the western boundary current [34,35]. Circulation in the Bering Sea is strongly influenced by the Alaska Stream, which enters the Bering Sea through 14 passes along the Aleutian Archipelago, which acts as a porous boundary between the North Pacific and the Bering Sea [35]. Inflow into the Bering Sea is balanced by outflow through the Kamchatka Current such that circulation is a continuation of the North Pacific subarctic gyre, moving eastward along waters off the northern shore of the Alaska Peninsula (Fig 1). The relatively shallow (< 80m) and narrow (~30km) Unimak Pass, near the documented western limits of native eelgrass distribution in Alaska, forms the only significant conduit between the shelves of the Gulf of Alaska and the EBS-LMEs, permitting a strongly northward seasonal flow of a portion of the Alaska Coastal Current. Flow through the pass is predominantly baroclinic, with speed of northward transport maximizing in the fall and winter and minimizing in the late spring and summer. Eddies are also ubiquitous in the Bering Sea; these are often anticyclonic in both the eastern and western side of the basin, and may facilitate localized deviations from net ocean current flow.

Ocean currents, oceanographic mixing patterns, geographic barriers, and distance all influence eelgrass distribution and apparently play a role in the differentiation of *Z*. *marina* populations elsewhere [20,21,36–39]. Muñiz-Salazar et al. [38] suggest the 1300-km long Baja California peninsula and varying oceanographic mixing patterns act as barriers limiting gene flow among eelgrass populations in the southwestern-most limits of the species’ range along the Pacific coast of North America and Gulf of California. Although seed rafting on floating reproductive shoots may be an effective form of dispersal for eelgrass [40,41], dispersal of both pollen [42] and seeds [43] is typically limited to just a few meters even in areas with strong tidal currents. Thus, the species is expected to show significant interpopulational structuring. Further, extensive vegetative reproduction through branching of rhizomes and formation of shoots is expected to limit genetic diversity within local subpopulations and amplify genetic structure between populations. Although flowering response apparently increases at the extremes of *Z*. *marina*’s range when compared to temperate populations, northern latitude populations along the North Pacific coast of North America flower less frequently than at southern latitudes [44]. Thus, levels of population differentiation likely increase latitudinally, while diversity will likely decrease, as observed in other plants [45] (although see [37] for disparate signals in eelgrass of the North Atlantic). Given ocean currents play an important role in the movement of gametes and individuals between populations, gene flow between discrete populations as assayed using a coalescence approach [46,47] should correspond to net movement of ocean currents.

We expect eelgrass populations in high-latitude habitats to show lower levels of genetic diversity than southern populations, and be significantly partitioned at the interpopulational level. Further, at least regionally, prevailing direction of gene flow should correspond to ocean current patterns, which differ between the two LMEs separated by the Alaska Peninsula and Aleutian Island archipelago. However, unlike for peninsulas elsewhere on the Pacific coast of North America that may sharply restrict or prohibit gene flow among eelgrass populations [38], the boundary between the EBS- and GoA-LMEs may be relatively semipermeable for eelgrass, particularly at False Pass, east of Unimak Island where a portion of the Alaska Coastal Current flows northeastward into the Aleutian North Slope Current. Thus, it is unclear whether the Alaska Peninsula, as well as different current velocities and mixing regimes in the EBS- and GoA-LMEs, have constituted an effective historical dispersal barrier for this species, as have similar landscape features and ocean currents elsewhere in North America [38], and whether interregional structure is present, as in some other marine species in the region [48–50].

The central aim of this study is to characterize genetic structure in largely undisturbed native populations of *Z*. *marina* along the northwestern-most Pacific coast of North America. Using nuclear microsatellite markers and nucleotide sequence information from the chloroplast maturase K gene (*mat*K) and the nuclear 5.8S rRNA gene and associated internal transcribed spacers, ITS-1 and ITS-2, we: (i) validate species, given plasticity in morphology within *Zostera* can confound species determinations [16,51] leading to erroneous assessments of genetic diversity and differentiation; (ii) quantify levels of genetic diversity and structuring within and among natural undisturbed populations of *Z*. *marina* along the North Pacific and Bering Sea coasts of Alaska, which are geographically separated by the Alaska Peninsula; (iii) test for association between genetic and geographical distances among populations within the two regions, and (iv) test for polarity of gene flow among populations within the EBS- and GoA-LMEs. Because the population genetic structure of marine angiosperms, as in other species, has been influenced by Pleistocene environmental events [52], and because ice-free regions in the high latitude Pacific have been hypothesized as Last Glacial Maximum (LGM) refugia for eelgrass and a source of colonization for North Atlantic populations [37], we also (v) test for genetic signatures consistent with the presence of LGM refugia in these high latitude Northeast Pacific region populations.

## Materials and Methods

### Ethics Statement

All eelgrass samples collected in United States waters were obtained on public access lands, and all researchers sampling in United States waters, including California, received permission for sampling eelgrass from the appropriate state regulatory agencies where required (e.g., California Department of Fish and Game, Washington Department of Natural Resources, Alaska Department of Fish and Game). Eelgrass samples from Yaquina Bay were obtained on public access lands and no permission was required because the plant species is not endangered or protected in the area sampled. In Alaska, permission was also obtained from the specific U. S. Fish and Wildlife Service Refuges for sampling on Refuge lands. Samples in Mexico were collected with permission from SEMARNAT (Secretaria de Medio Ambiente y Recursos Naturales), the federal agency responsible for promoting the protection of natural resources in Mexico and the regulatory authority for collecting in Mexico’s intertidal areas. The species is not considered endangered or protected in areas where the sampling occurred in waters of either the United States, or Mexico.

### Study sites

**Fig 2:**
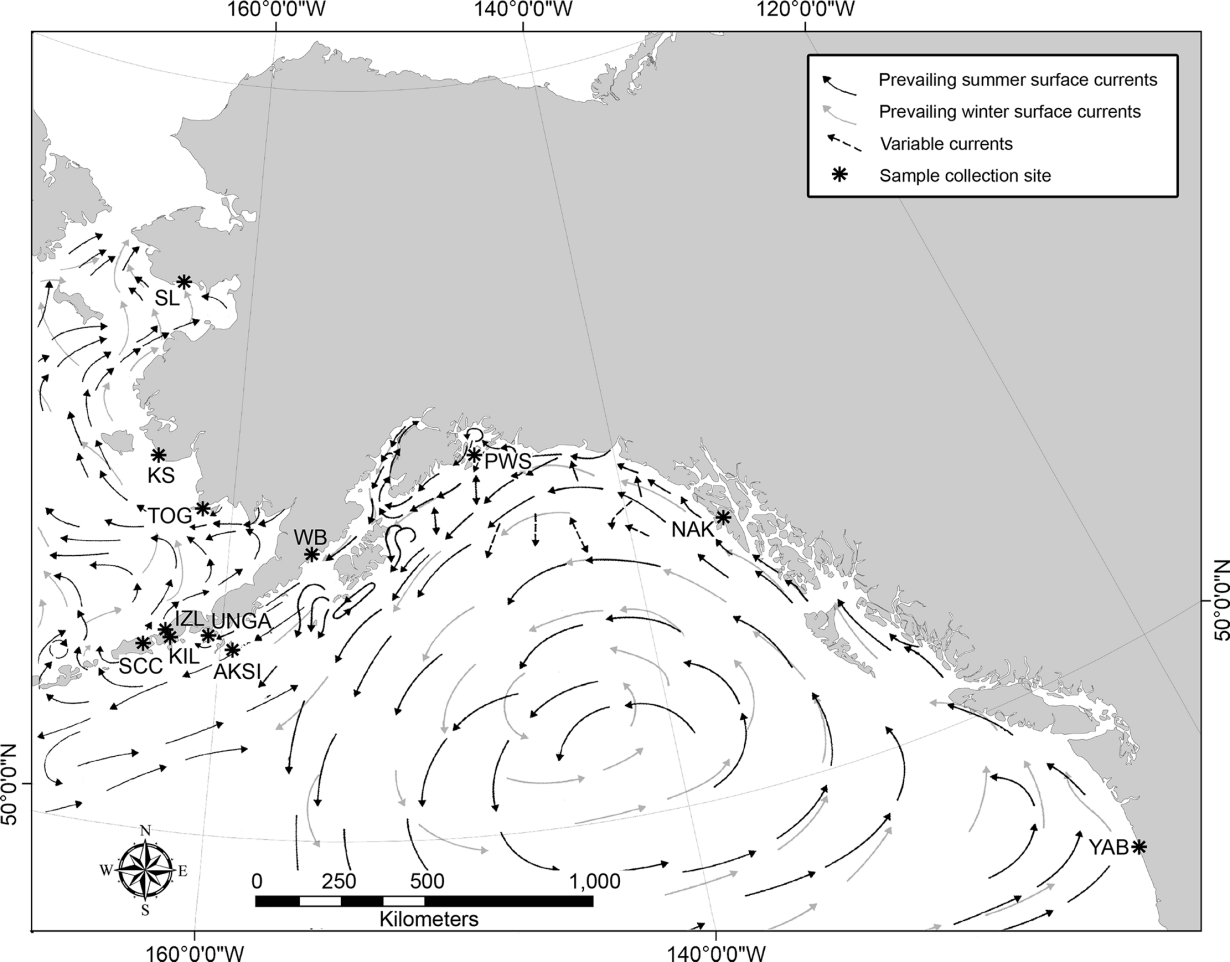
Sites sampled for *Z*. *marina* analyzed in this study, with respect to surface currents. Sample locales for *Z*. *marina* are shown with asterisks (*). Arrows indicate direction of prevailing seasonal surface currents derived from Brower et al. [53,54]. The base map was produced using ArcGIS version 10.2.2 [32].

Eelgrass meadows from 12 sites along the mid-to-northern Pacific Coast of North America were sampled during summer months between 2000–2006 (Fig 2). Eelgrass meadows at Safety Lagoon (SL) on the Seward Peninsula, Saint Catherines Cove (SCC) and Izembek Lagoon (IZL) on the northern side of the Alaska Peninsula, and locales in the Togiak Bay (TOG) area inhabit geographically separated coastal lagoons on the southern and eastern coast of the Bering Sea. Eelgrass from the Kuskokwim Shoals (KS) inhabits small embayments associated with barrier islands forming the Kuskokwim Shoals in the northern Kuskokwim Bay. Meadows in Kinzarof Lagoon (KIL) and Wide Bay (WB) occur in geographically isolated coastal lagoons along the Pacific coast side of the Alaska Peninsula. Eelgrass collected from Unga (UNGA) and Simeonof (AKSI) islands of the Shumagin Island group, south of the Alaska Peninsula, are from beds found in sheltered bays along the open coast environments. Eelgrass from Montague Island (PWS) in Prince William Sound in south central Alaska, Nakwasina Bay (NAK) in the Alexander Archipelago of southeastern coastal Alaska, and from Yaquina Bay, Oregon (YAB), occupy sheltered bays along the northwestern Pacific coast of North America. Hereafter, locales from which these samples were collected are referred to as ‘populations.’

At all but one study site (KS), eight to sixty individuals of *Z*. *marina* separated by 20–200 m were randomly collected during low tides. For KS, > 250 samples were retrieved from an outboard engine propeller at various sampling sites along a general transect of 2.5 km. Plants were scraped clean of visible epiphytes and invertebrates, blotted dry with paper towels and stored in powdered silica gel until DNA was extracted from leaf tissue. All individuals in each population were sampled within an area smaller than 750,000 m^2^. This area is much larger than the genetic neighborhood area (500–1600 m^2^) for eelgrass suggested by Ruckelshaus [42,55] and Reusch et al. [56] but is nevertheless appropriate for examining large-scale (phylogeographic) population differentiation [38].

### Laboratory analyses

See S1 Methods for details of laboratory analyses. Following extraction and quantification of DNA, nucleotide sequence data from 348 basepairs (bp) of the *Zostera* chloroplast maturase K (*mat*K) gene and 488 bp of the 5.8S rRNA gene and associated internal transcribed spacers, ITS-1 and ITS-2 (ITS) were collected following methods presented in the literature [16]. Sequence data were collected from 3–12 samples at the 11 locales along the coast of Alaska. For comparative purposes, we also obtained sequence data from *mat*K and ITS from 3–13 individuals per population (except Morro Bay for ITS) in the other two Pacific Coast LMEs of North America: 1) the California Current LME (CC-LME), including Puget Sound, Washington (PS), Monterey (MON), Humboldt (HUM), and Morro bays, California, and four lagoons along the coast of Baja California Peninsula (Estero Punto Banda, Bahia San Quintin, Laguna Ojo de Liebre, Laguna San Ingnacio [38], and three lagoons in the 2) Gulf of California LME (GoC-LME: Bahia Concepcion, Punta Chueca, and Isla Tiburon [38]). All *mat*K and ITS data were compared with data from prior research [16] and sequences accessioned in GenBank. Fragment data were collected at 10 polymorphic microsatellite loci [56,57]) from the 12 locales in Alaska, following procedures outlined elsewhere [38,58]. For comparative purposes, genotypes generated from YAB (see Muñiz-Salazar et al. [38]) were also included in analyses.

### Data analyses

#### Analyses of sequence data

*Mat*K and ITS sequences were collapsed into haplotypes using DNACollapser [59]. To address proposals that more than one species of *Zostera* occurs in Alaska and to verify that our analyses were from populations of *Zostera marina*, we used the BLASTN algorithm [60] to compare all *matK* and ITS haplotypes to data from different *Zostera* species deposited on NCBI (see [16,61,62]). As well, sequences were compared to homologous information from both loci obtained from accessioned specimens used by Talbot et al. [16] to represent North Pacific *Z*. *marina*: UAM Herb:43460, collected from Wide Bay, Alaska Peninsula (GoA-LME); and UAM Herb:40475, collected from Cowpack Lagoon on the Seward Peninsula (EBS-LME). We note that UAM Herb:40475, previously determined to be *Z*. *marina*, was annotated and ascribed in 2006 to *Z*. *angustifolia*. Unfortunately, no sequence data for ITS or *mat*K attributed to *Z*. *angustifolia* are archived in GenBank. Nevertheless, we assumed that if two different *Zostera* species occupied habitats within the two high latitude LMEs, and *Z*. *angustifolia* was indeed a discrete lineage and one of those species, we would observe 1) diagnostic differences between UAM Herb: 40475 (cf. *Z*. *angustifolia*) and UAM Herb: 43460 (*Z*. *marina*) at the ITS and *mat*K genes, and 2) among sequences assayed from individuals representing our target populations. Further, 3) if sampled ‘populations’ actually comprised individuals from different species, we should observe significant linkage disequilibrium (and likely deviations from Hardy Weinberg proportions) across most or all microsatellite loci collected across individuals sampled from within the mixed ‘population’ [63].

An unrooted phylogenetic network was constructed for *mat*K data using NETWORK Version 4.612 (Fluxus Technology Ltd., fluxus-engineering.com; [64]). Homologous sequence from three other species of *Zostera* (*Z*. *asiatica*: AB125360; *Z*. *japonica*: AB125361; and *Z*. *caulescens*: AB125358; [62]) were included for comparison.

#### Clonality and genetic diversity

Details of analyses to assess levels of genetic diversity at microsatellite loci are provided in S1 Methods. To determine the level of clonality and generate a dataset for subsequent population genetics analysis, we employed the match statistics option in Microsatellite Toolkit [65] to identify samples sharing identical multilocus genotypes, presumably representing a single clone, among the samples, and GenClone ver. 1.0 [66], to estimate genotypic richness (R), a measure of clonality within the population [67]. Populations with a value of R at unity have 0.0% clonality; lower values of R indicate higher levels of clonality. Following assessment of R, the dataset was pruned by eliminating data from all but one sample representing a genetic individual (a genet). This pruned dataset was used to test the power to detect individuals by calculating the probability of observing identical multilocus genotypes between two individuals sampled from a population (P_ID_ and P_IDsib_; [68]), using the program GIMLET v. 1.3.2 [69], and to conduct subsequent population genetics analyses. We quantified genetic variation (using data from genets only) and tested for neutrality in microsatellite loci using a variety of computer programs routinely used to analyze genetic data [70,71]. In all cases of multiple tests, significance levels were adjusted using sequential Bonferroni corrections [72].

#### Population structure

Details of analyses to assess levels of population and regional structure are provided in S1 Methods. Significance of spatial variation in microsatellite allele frequency between populations was assessed using F-statistics and their analogs (***θ***, [73]; **ρ**, [74,75]), employing several computer programs routinely used to analyze genetic data [76–78]. Significance of tests based on random permutation of alleles between populations, and p-values were adjusted using Bonferroni corrections. We also tested for differences in the distributions of alleles and genotypes across populations using a log-likelihood (G) based exact test [79], using GENEPOP’007 [80] and judging the significance after applying sequential Bonferroni procedures.

To further investigate the pattern of population structuring, we also applied a Bayesian clustering approach [81]. Data were analyzed to detect the occurrence of population structure, without a priori knowledge of putative populations, under two situations: situations under which i) the number of clusters was pre-defined (K = 2, corresponding to the two LMEs, and K = 3–4, based on results of the regional analyses of molecular variance; see below); and ii) the number of clusters was not predetermined, but with an upper limit of 20 (more than the sampled locales), to facilitate the identification of more detailed patterns across the geographic distribution. Each Bayesian clustering analysis was repeated 10 times to ensure consistency across runs. We also investigated genetic structuring and visualized regional and between-population by conducting discriminant analyses of principal components (DAPC), using the R package *adegenet* [82,83]. Optimal cluster number within each region and population was determined using sequential k-means algorithm on principal components and discriminant functions transformed data and compared to original population identification, with probability of membership in each population determined for each sample [84].

#### Regional structure

We employed hierarchical analyses of molecular variance (AMOVA) to test the hypothesized regional relationship between the GoA-LME and EBS-LME populations relative to a suite of alternative hypotheses, quantifying variance at four hierarchical levels: 1) between regions (EBS-LME and GoA-LME), 2) among populations within regions, 3) among individuals within populations, and 4) within individuals. Statistical significance of variance measures was assessed via non-parametric permutation [77].

#### Population relationships and tests of isolation-by-distance

We examined genetic relationships among populations by creating a neighbor-joining tree [85], inferred from allelic frequency data using Cavalli-Sforza and Edwards’ [86] chord distances (D_CE_), evaluating confidence of the tree topology via bootstrapping over loci (1000 replicates) using POPULATIONS 1.2.30 [87] and visualizing the resulting networks using TREEVIEW Ver. 1.6.6 [88]. We used IBD 3.0 [89] to compare pairwise genetic and geographic distances with those expected under a stepping-stone model of population structure [90], testing for a correlation between the logarithm of the geographic distance and Rousset’s genetic distance [91] for populations overall and within each region, separately. Reduced-major-axis regression implemented in the program IBD was used to determine the slope of significant regression for the Pacific coast and Bering Sea graphs.

To determine whether there is an inverse relationship between genetic diversity and latitude, as expected if contemporary populations of eelgrass in the North Pacific colonized from temperate or southern refugia following climate amelioration at the Pleistocene/Holocene boundary, we performed a linear regression analysis fitting two commonly-used measures of genetic diversity: expected heterozygosity (H_E_) data and standardized allelic richness (AR [92,93]), to degrees latitude. An inverse relationship between genetic diversity and latitude is expected if genetic diversity decreases constantly along a latitudinal gradient following an isolation-by-distance pattern. We combined data from this study (YAB, NAK, WB, IZL, TOG, SL only) and comparable data from other populations (Baja California: Bahia Magdalena, Bahia San Quintin, Estero Punto Banda, [38]; California: MON; and Washington: PS, represented by Shallow Bay in [58]). Data for six (CT-3, CT-20, GA-1, GA-2, GA-3, and GA-5) of the 10 loci used in this study were available for all 12 populations (http://dx.doi.org/10.5066/F7GQ6VTK), and all H_E_ values were derived from genets.

#### Gene flow rates and polarity

We estimated the magnitude and polarity of gene flow among populations within the two regions using the maximum likelihood approach implemented in MIGRATE 2.0.3 [47,94]; details of the gene flow analyses are provided in S1 Methods. MIGRATE uses a coalescence approach to estimate gene flow rates (N_*m*_) among populations, assuming a constant per-locus mutation rate (μ). To test whether net current direction can predict localized direction of gene flow, we performed MIGRATE analyses separately for Bering Sea and Pacific coast populations, estimating full models, Θ = 4N_e_μ (the composite measure of effective population size and mutation rate; see S1 Methods) and all pairwise migration parameters individually from the data and comparing them to a restricted island model whereby Θ and pairwise migration parameters are constrained to be equal between populations. Competing models were evaluated for goodness-of-fit given the data using a log-likelihood ratio test. The resulting statistic from the log-likelihood ratio test is equal to a χ^2^ distribution, with the degrees of freedom equal to the difference in the number of parameters estimated in the two models [94].

## Results

### Nuclear and chloroplast DNA sequencing

Three-hundred forty-eight bp of the *mat*K gene and 488 bp of the ITS genes were recovered from among 170 and 103 sequenced samples, respectively, from EBS-LME to the GoC-LME. Combined with data from [16], a total of 178 and 118 samples were used to make inference from these gene region, respectively (S1 Fig). Sequence data obtained from samples collected from Alaska (n = 75 and 56, respectively, for *mat*K and ITS) demonstrated we sampled and extracted genomic DNA from *Z*. *marina* and not the invasive *Z*. *japonica*, which may occur in Alaskan coastal waters (i.e. UAM:Herb:248738, 248740, 248741, and 248739). We observed two ITS haplotypes (S1 Fig) among all samples assayed from Alaska through Baja California and the Gulf of California. A single ITS variant (ITS1) was observed within all populations in Alaska (NAK through SL; see S1 Fig), including accessioned voucher specimen UAM Herb 43460. ITS1 shared 99% sequence homology (a single site G ↔ A transition) with sequences from *Z*. *marina* reported by Les et al. [95] (AY077986, vouchered at CONN: Yarish s.n.), and differed at 98 nucleotide sites (46 insertions/deletions, 21 transitions and 30 transversions) from *Z*. *japonica*, reported in Talbot et al. [16]. Further, ITS1 sequences were identical to homologous sequences from other individuals sampled from all other North Pacific North American *Z*. *marina* samples (PS, YAB, HUM) assayed, as well as in Japan (S1 Fig). However, a novel variant (ITS2) co-occurred with ITS1 in Baja California (the CC-LME) and GoC-LME (S1 Fig). The novel variant, present in 28% of samples assayed from Baja California and Gulf of California locales (S1 Fig), differed from the common North American variant at a single transition (G ↔ A) change. Sequences have been deposited in GenBank (KU704817-KU704920).

**Fig 3:**
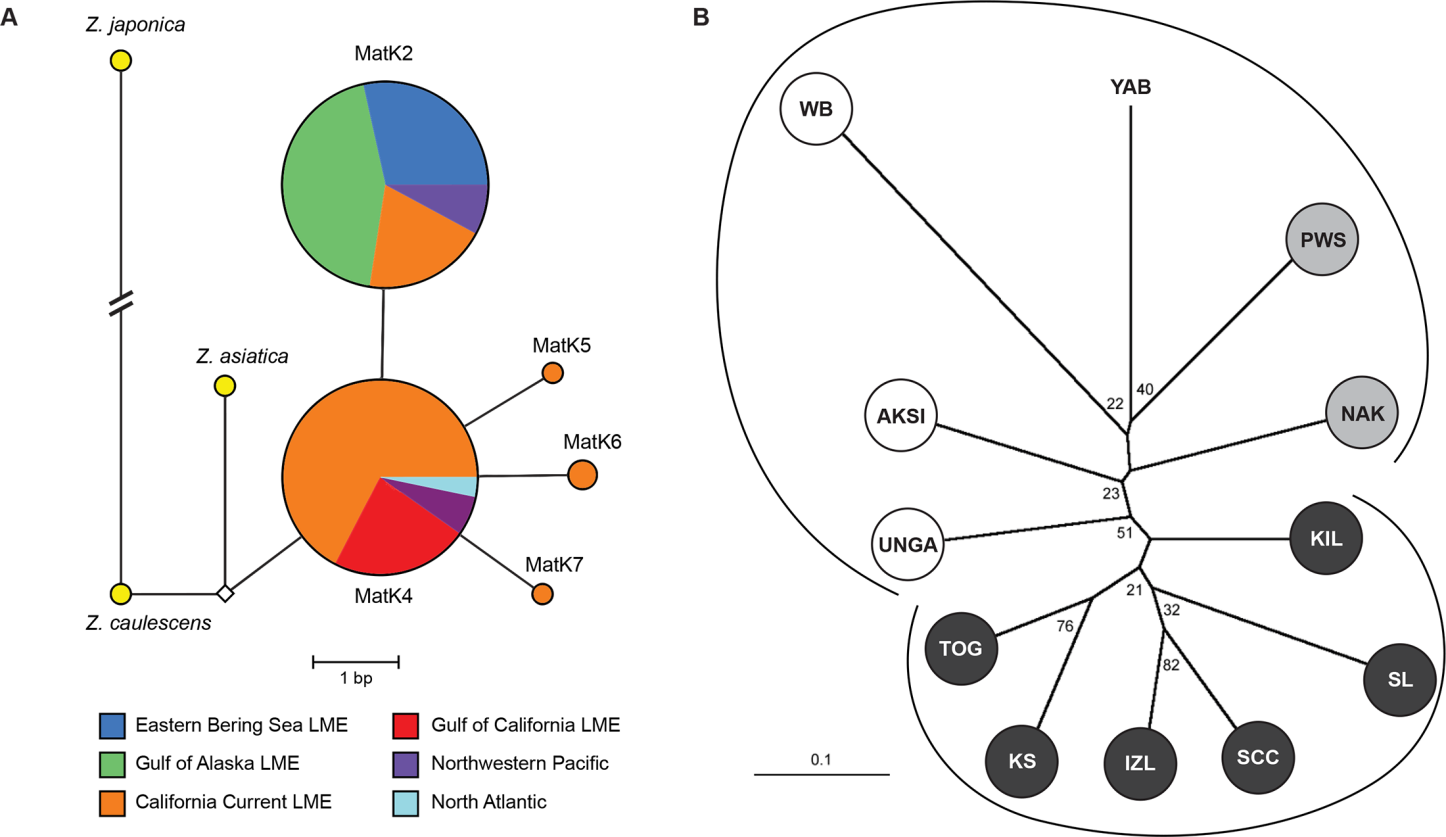
**(A) Parsimony network of chloroplast *mat*K haplotypes from *Z*. *marina* and (B) a neighbor-joining tree illustrating relationships among Pacific coast populations of *Z*. *marina*.** (A) A parsimony network of chloroplast *mat*K haplotypes assayed from *Z*. *marina* from the EBS-LME (n = 29), GoA-LME (n = 45), CC-LME (n = 86), and GoC-LME (n = 21). Data are from populations listed in S1 Fig, along with homologous data reported elsewhere [62,96] for samples collected from various locales within the CC-LME (GenBank accessions: *mat*K2—EF19834, AB125354, AB096164; *mat*K4—EF1983339, EF198342, AB125356, AB125355). Homologous data from *Z*. *marina* from the Northwest Pacific (Japan and Korea; n = 14, data from [16,62]), the North Atlantic (Germany, n = 2, GenBank accessions: JN225374-75; Ukraine, n = 1, GenBank accession: JQ990925), and one sample each from *Z*. *japonica* (GenBank accession: AB125361), *Z*. *caulescens* (GenBank accession: AB125358), and *Z*. *asiatica* (GenBank accession: AB125360) are included for comparison. The size of the node corresponds to the frequency of each haplotype within each LME or region, and length of branch corresponds to number of changes, unless noted with diagonal slant bars. The small white diamond indicates an intermediate ancestral allele that was not sampled. (B) Neighbor-joining tree illustrating relationships among Pacific coast populations of *Z*. *marina*. D_CE_ distances were generated by data from 10 microsatellite loci. Bootstrap values (2000 replications) are listed at the node. Black, white, and gray circles identify populations belonging to the three discrete groupings (Model D) uncovered via AMOVA analyses to reflect significant and highest allelic variance at the regional level (see Results, in text, and S1 Table).

Five *mat*K haplotypes (*mat*K2, 4, 5, 6, 7), which differed at up to two segregating sites across four variant nucleotide sites (3 transitions, 1 transversion), were observed across the species’ North American Pacific coast distribution (S1 Fig and Fig 3A). Similar to ITS, all Alaskan populations, including UAM 40475 (ascribed to *Z*. *angustifolia*), were characterized by a single *mat*K variant (*mat*K2; see S1 Fig). *Mat*K2 was also found in North Pacific samples north of 40° latitude, including PS, YAB, and HUM, as well those reported for *Z*. *marina* by Tanaka et al. [61] (GenBank accession: AB096164; accessioned at TNS: Tanaka 99190) and Kato et al. [62] (Zmm1, GenBank accession: AB125354), but not by us (or other researchers, to date) at latitudes lower than 40° along the north Pacific coast of North America, including Morro Bay, MON, or Baja California and GoC-LME populations. *Mat*K4, the most common variant in the CC-LME, was observed in PS and HUM, but not in YAB; this variant also occurred in the GoC-LME (S1 Fig and Fig 3A). Laguna Ojo de Liebre on the Pacific coast of Baja California possessed three *mat*K variants (S1 Fig). Haplotype sequences have been deposited in GenBank (KU704921-KU705084).

### Microsatellite Analyses

#### Clonality and genetic diversity

There were 61 pairwise instances in which we found that more than one sample yielded an identical 10-locus genotypes (seven instances in YAB, six in UNGA, 46 in PWS, and one each in AKSI and TOG). In no instance were identical genotypes observed in samples from across two or more populations. Genotypic richness was lowest in UNGA and PWS (Table 1). We dropped all but one of any set of samples that shared identical 10-locus genotypes, assuming the set represented a clone, since the probability of observing identical multilocus genotypes in two unrelated genets sampled from populations in these regions averaging 1/3,346,720, and ranging from 1/674 within KS to 1/29,533,372 within YAB (Table 1). Microsatellite genotype data are accessioned at the USGS Alaska Science Center data repository (http://dx.doi.org/10.5066/F7GQ6VTK).

**Table 1:** Measures of genetic diversity at ten microsatellite loci for *Zostera marina* populations along the Pacific coast of North America.

POPULATION	R	n	P	A	AR	PA	H_O_	H_E_	P_ID_	P_IDsib_
*Eastern Bering Sea LME*										
Safety Lagoon (SL)	1.00	34	0.80	5.4	2.49	5	0.29	0.31	8.620e-05	3.137e-02
Kuskokwim Shoals (KS)	n/a	29	0.60	2.6	1.95	0	0.18	0.21	1.484e-03	9.107e-02
Togiak (TOG)	0.97	49	0.80	6.2	2.59	3	0.20	0.23	6.276e-05	6.419e-02
Izembek Lagoon (IZL)	1.00	71	0.90	7.4	2.77	4	0.32	0.33	3.275e-05	2.032e-02
Saint Catherine Cove (SCC)	1.00	24	0.90	4.7	2.67	0	0.33	0.36	2.952e-05	1.825e-02
*Gulf of Alaska LME*										
Kinzarof Lagoon (KIL)	1.00	48	1.00	7.6	3.10	2	0.34	0.36	1.666e-06	1.547e-02
Wide Bay (WB)	1.00	33	0.90	4.1	2.41	2	0.32	0.33	5.816e-05	2.435e-02
Unga Island (UNGA)	0.56	6	0.70	2.2	2.20	0	0.35	0.31	1.621e-05	4.609e-02
Simeonof Island (AKSI)	0.83	6	0.80	2.9	2.90	0	0.33	0.33	1.203e-08	3.224e-02
Montague Island (PWS)	0.67	46	1.00	7.0	3.29	5	0.46	0.45	5.574e-07	5.438e-03
Nakwasina Bay (NAK)	1.00	30	0.90	5.6	2.84	2	0.35	0.35	2.608e-06	1.970e-02
Yaquina Bay (YAB)	0.80	25	1.00	6.0	3.62	4	0.60	0.56	3.386e-08	1.895e-03

Values are as follows: R = clonal diversity (genotypic richness), n = sample size for analysis (genets only); P = percent polymorphism, A = average number of alleles per locus, AR = allelic richness [92,93]; PA = number of private alleles; H_O_ = observed heterozygosity; H_E_ = expected heterozygosity [97]; P_ID_ = probability of identity given a randomly breeding population; P_IDsib_ = probability of identity given populations are comprised only of first-order relatives. All values except R were calculated using genets only.

Number of alleles detected per locus ranged from four (CT-19, GA-4) to 43 (CT-17) and the mean number of alleles per locus per population (observed allelic diversity) ranged from 2.2 to 7.6 (Table 1). All EBS-LME populations and four of the seven GoA-LME populations were monomorphic (*P <* 1.0) for at least one locus; only KIL, PWS and YAB were polymorphic at all loci (Table 1). Allelic richness, calculated based on the sample size at UNGA, varied from 1.95 at KS to 3.62 at YAB (Table 1); allelic diversity was on average higher in GoA-LME populations (mean allelic diversity = 2.91) than EBS-LME populations (mean allelic diversity = 2.49). Except in KS, SCC, UNGA and AKSI, private alleles occurred in all populations, and the greatest number of private alleles (5) was found in SL and PWS (Table 1). Overall, the EBS-LME populations demonstrated the highest number of private alleles. Average H_E_ within populations ranged from 0.21 at KS to 0.56 at YAB; average H_O_ ranged from 0.18 at KS to 0.60 at YAB (Table 1). Overall H_E_ was 0.46; average H_E_ was higher in GoA-LME (0.35) than EBS-LME populations (0.29). Overall H_O_ was 0.33; again, average H_O_ was higher in GoA-LME (0.35) than EBS-LME (0.26) populations.

The global test revealed significant departures from HWE in 2 of the 12 populations: SCC (*P =* 0.003) due to significant heterozygote deficit at one locus (CT-3; *P =* 0.0013), and TOG (*P <* 0.0001) due to significant heterozygote deficit at two loci (CT-3, *P =* 0.0134; GA-5, *P =* 0.0001). No significant inbreeding values were observed across loci overall, after correction for multiple tests (F_IS_ = -0.154–0.115, *P <* 0.0004 for all values). Exact tests among the 382 possible pairwise comparisons demonstrated significant linkage disequilibrium (*P <* 0.005) in 15 cases (four pairwise comparisons within YAB, three within WB, two within SCC, SL, PWS, and TOG, one within KIL). This is lower than the number of spurious results expected for 382 pairwise comparisons (19.1), although more significant pairwise comparisons were observed (4) than expected spuriously (2.25) within YAB. The global test across populations, for all 45 pairings of loci, was not significant (*P >* 0.087). Given the general adherence to HWE expectations and lack of significant linkage disequilibrium, coupled with the results of the sequence analysis of chloroplast and nuclear loci, we conclude that our populations are comprised of *Z*. *marina* and do not represent either *Z*. *japonica*, or *Z*. *angustifolia*. All data from genets and across loci were retained for analysis of population structure.

#### Population and regional structure

We observed significant differentiation based on variance of allele frequency overall (***θ*** = 0.302, *P <* 0.0001); values of **ρ** were higher (**ρ** = 0.366, *P <* 0.0001). Pairwise population differentiation estimated by θ_ST_ among all pairs of populations ranged from 0.043 to 0.592 (S1 Table). Variance in allele frequency between populations (***θ***_*ST*_) was significantly different from zero among all North Pacific coast population pairwise comparisons (excluding YAB) except one: NAK–AKSI (S1 Table); **ρ**_*ST*_ values were significant across all but nine pairwise comparisons, all involving comparisons with AKSI or UNGA, the populations represented by the smallest population sizes (Table 1, S1 Table). Average ***θ***_*ST*_ values were similar across EBS-LME (***θ***_EBS_ = 0.225) and North Pacific (GoA-LME populations, and YAB (***θ***_NPacific_ = 0.264), but overall **ρ** was higher in the EBS-LME populations **ρ**_EBS_ = 0.359; **ρ**_NPacific_ = 0.322). In 37 pairwise comparisons, **ρ**_*ST*_ values were greater than ***θ***_*ST*_ values; there were 29 instances of the reciprocal (S1 Table). The greatest ***θ***_*ST*_ (0.592) value among all North Pacific Alaskan populations occurred between WB and KS; the greatest **ρ**_*ST*_ value (0.795) was between KS and SCC (S1 Table). Significant differences in the distribution of alleles were observed overall (χ^2^ = ∞, *P <* 0.0001) and for all pairwise population comparisons (χ^2^ = 43.928 - ∞, df = 18–20, *P <* 0.0001), except AKSI and UNGA (χ^2^ = 28.564, df = 18, *P =* 0.0540).Results of the hierarchical AMOVA (S2 Table) indicated that only 6.30% (***θ***_p_ = 0.063, *P* = 0.022) of the total genetic variation could be attributed to variation between the two biogeographical regions (GoA-LME vs. EBS-LME), while 25.2% (***θ***_s_ = 0.252; *P <* 0.0001) of the variance could be explained by differences among populations within the two regions (S2 Table). Among the eight alternative models, ***θ***_p_ became significant when KIL was included in the same grouping as IZL and SCC (Models C, D, and H; S2 Table), with ***θ***_p_ maximized in Model D, in which an eastern and western component of the North Pacific LME was assumed. However, while AMOVA analyses based on the assumption of SMM also found variance to be significantly partitioned among populations within regions for all models (e.g., **ρ**_s_ = 0.304, *P <* 0.001 for Model D), **ρ**_p_ was not significant for any between-region test (e. g., **ρ**_p_ = 0.195, *P =* 0.054 for Model E). Bayesian clustering confirmed trends of significant population structuring observed with pairwise θ_ST_ comparisons: when analyses were not constrained to number of clusters, the highest posterior probability was obtained for K = 9 (Ln(X/K) = -7670.6, Ln*P =* 1.0; S2A Fig). When constraining cluster number to K = 2, Bayesian analyses confirmed AMOVA trends: all of the GoA-LME populations clustered together except KIL, for which the majority of individuals clustered with the EBS-LME populations; approximately half of the individuals within AKSI also clustered with the EBS-LME populations (S2B Fig). Constraining cluster number to K = 3 and K = 4 partitioned out WB as a unique population overall (K = 3) and within the North Pacific (K = 4), and highlighted increasing levels of admixture within GoA-LME locales at the eastern end of the Alaska Peninsula (S2C and S2D Fig).

**Fig 4:**
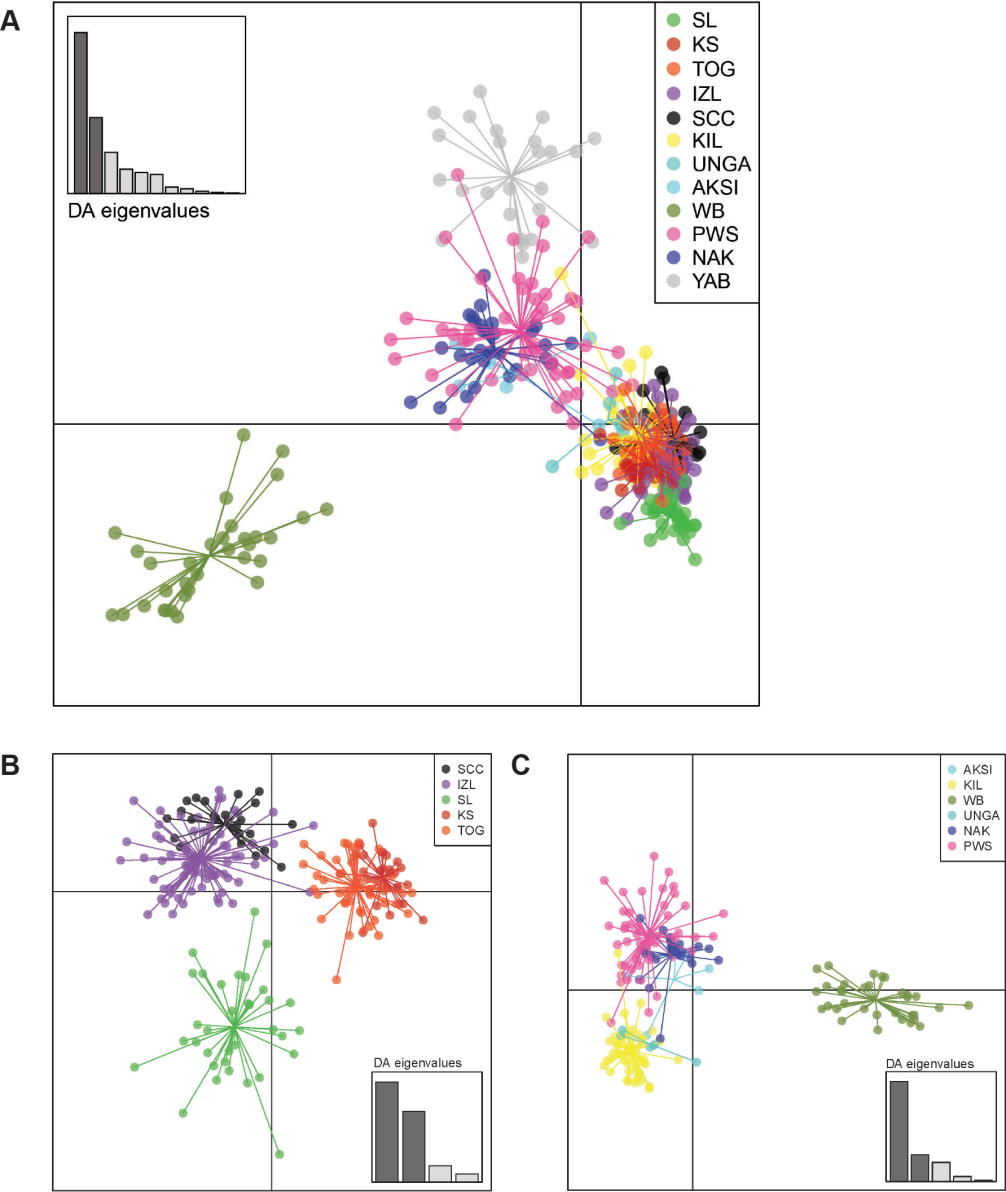
DAPC scatterplots from *Z*. *marina* populations in the EBS- and GoA-LMEs. (A) DAPC scatterplot showing genetic variation in all eelgrass populations from both the EBS-LME and GoA-LME. (B) DAPC scatterplot of genetic variation among EBS-LME populations only. (C) DAPC plot of genetic variation among GoA-LME populations only. The first two discriminant functions represent the majority of the genetic variation in each dataset, including 86.5% of the genetic variation among all populations, 91.8% among EBS-LME, and 82.5% among all GoA-LME populations.

Using the K-means algorithm as implemented in *adegenet*, the optimal number of clusters in the principal components transformed microsatellite dataset was 15 (BIC = 243.92), however this model did not perform significantly better than the null model of 12 populations (BIC = 246.30, LRT p-value = 0.19, df = 3), and in fact indicated 9–13, 17, and 20 cluster models did not perform significantly worse than the optimal model. Using 15 inferred clusters, YAB and WB were largely recovered as inferred populations 7 and 15, while other populations were divided among many inferred groupings with shared original sample populations, including SCC in 2, 4–6, and 8–10, KIL in 1, 4–6, 8–10, and 12 and IZL in 2, 4–6, and 8–10 (S3A Fig). Membership probability of samples assessed using DAPC also indicates distinctiveness of YAB and WB, while sample identity was largely shared among SCC, KIL, and IZL populations (S3B Fig). A scatterplot of the first two discriminant functions of the DAPC, representing 86.5% of microsatellite variation and 30 principal components, indicates EBS-LME populations cluster together, and overall show less genetic variation than GoA-LME populations, as suggested by tighter grouping of individuals in each population. GoA-LME populations, though still largely grouped together (except WB), show markedly more variation within populations than EBS-LME populations (Fig 4A), a pattern which remains clear when the first two discriminant functions of the EBS- and GA-LME populations are analyzed separately by region (Fig 4B and 4C), and represents 91.8% and 82.5% of the variation in the data, respectively. Number of principal components retained for the analysis had no significant impact on results, using number of principal components recommended by both α-scores and cross-validation methods [83].

#### Population relationships and tests of isolation-by-distance

The lowest pairwise genetic distances (D_CE_) occurred between populations in the EBS-LME (IZL–SCC: D_CE_ = 0.172); the largest occurred between a GoA-LME and EBS-LME population (WB–SL: D_CE_ = 0.558; complete data matrix not shown). The neighbor-joining(D_CE)_ tree revealed two main clusters (Fig 3B), supported by a slim majority (51%) of bootstrap replicates. One cluster comprises GoA-LME populations, and the other EBS-LME populations, with a single exception: KIL clustered more frequently with the EBS-LME populations of IZL and SCC.

We found no significant correlation between Rousset’s genetic [91] and geographic distances based on microsatellite data for the GoA-LME populations overall (r = 0.248, Z = 79.28, *P =* 0.161), or among populations within regions (GoA-LME: r = -0.095, Z = 20.68, *P =* 0.466; EBS-LME: r = 0.697, Z = 8.639, *P =* 0.064). However, significant correlations were observed between log-transformed genetic distances and geographic distances when all populations were pooled overall (r = 0.424, Z = -98.64, *P =* 0.045) and for populations within the EBS-LME (r = 0.873, Z = -15.12, *P =* 0.034).

**Fig 5:**
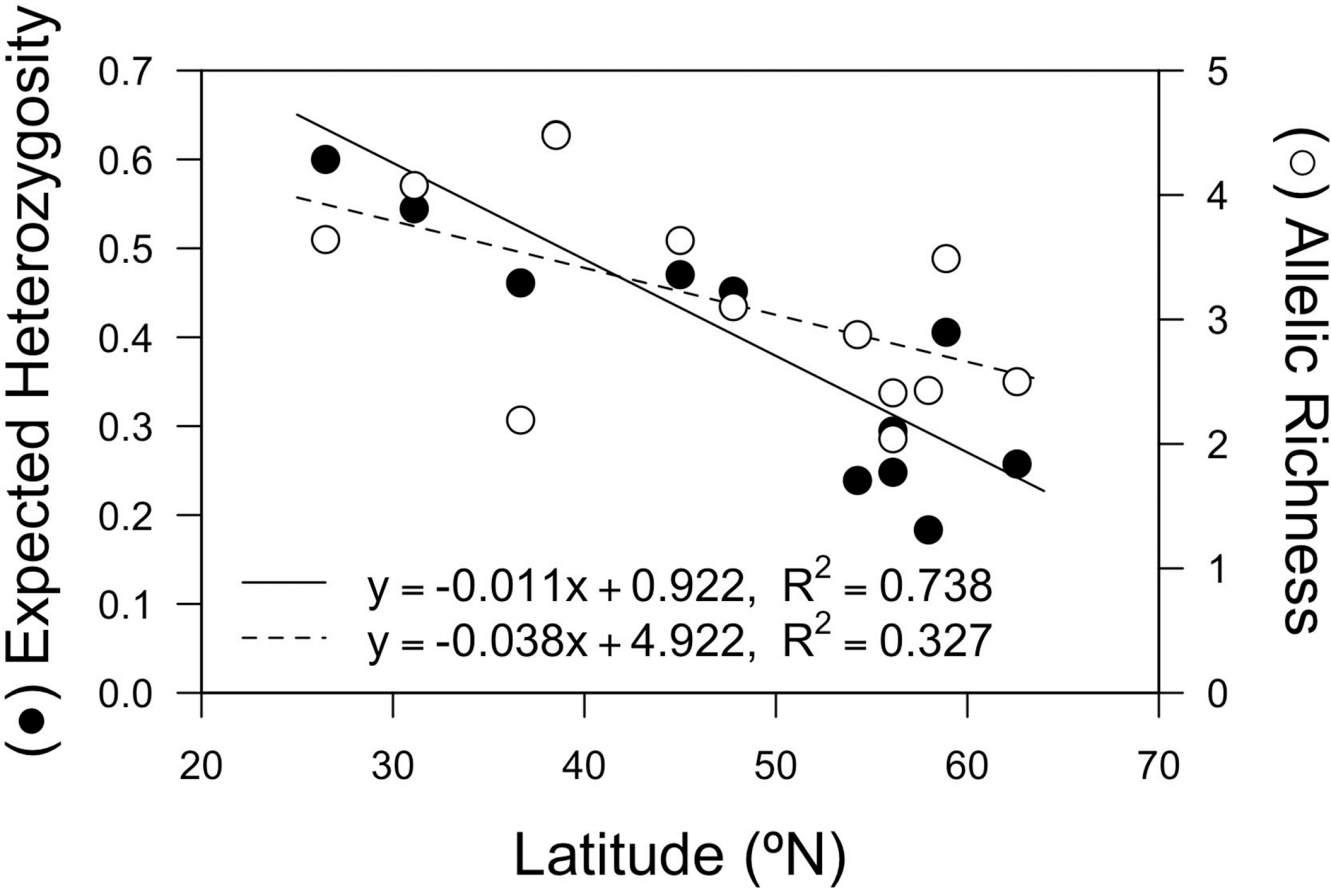
Relationship between genetic diversity and latitude. Mean expected heterozygosity (H_E_) and allelic richness (AR) for each of the 12 populations. Each point represents a population from this study or from previous studies [38,58]. Regression lines are shown for H_E_ (solid line) and AR (dashed line).

In the 12 populations distributed in a south-north latitudinal gradient, H_E_ and AR correlated inversely with latitude, explaining 73.8% (H_E_) and 32.7% (AR) of the variance in percentage (R^2^ = 0.738, *P <* 0.001; R^2^ = 0.327, *P =* 0.047, respectively; Fig 5). These results emphasize a decrease in genetic diversity in a southern to northern latitudinal gradient in eelgrass distribution along the north Pacific coast of North America.

#### Gene flow rates and polarity

**Fig 6:**
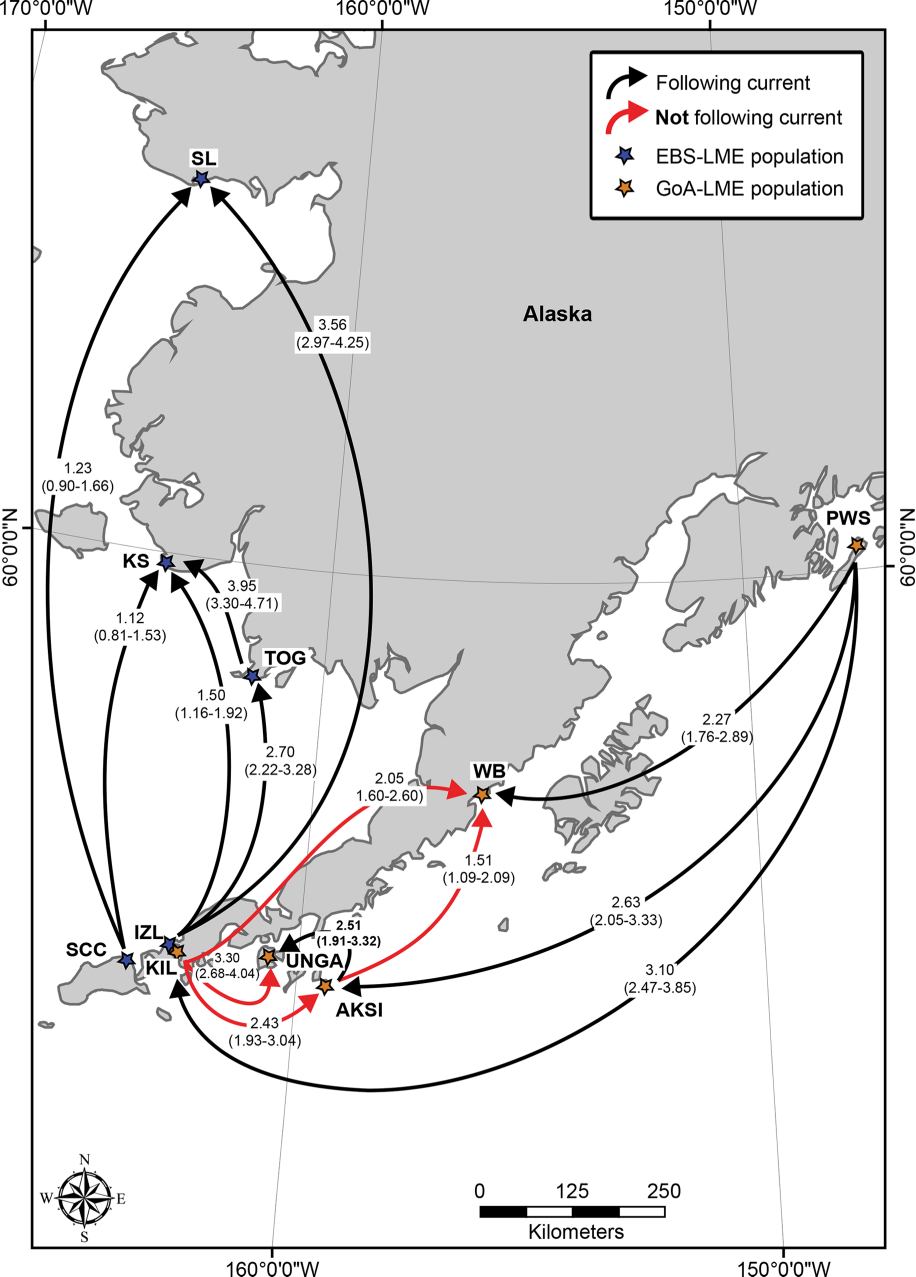
Schematic showing direction of instances of significant deviation from symmetry in interpopulational gene flow, based on MIGRATE analyses. Blue asterisks (*) signify EBS-LME populations; orange asterisks (*) signify GoA-LME populations. Black arrows indicate symmetrical gene flow concordant with surface current flow; red arrows show asymmetrical gene flow that contrasts with surface current flow. The base map was produced using ArcGIS version 10.2.2 [32].

We tested a 5-population geographic model for EBS-LME populations (SCC, IZL, TOG, KS, SL), and a 7-population geographic model for GoA-LME populations (YAB, NAK, PWS, AKSI, UNGA, WB, and KIL), and based on our inference of partial genetic partitioning between regions and to test for polarity associated with LME-specific current patterns. *N*_*e*_*m* and Θ values calculated in MIGRATE for Bering Sea populations ranged from 0.265–3.946 migrants per generation, with Θ ranging from 0.969–1.027, and for Pacific Coast populations ranged from 0.178–3.30 migrants per generation, with Θ ranging from 0.924–1.074 (S3 and S4 Tables). The full model (all parameters allowed to vary independently) had significantly higher likelihoods than the restricted island model (symmetric inter-population effective migrant rates and Θ) across all marker types, indicating asymmetric gene flow among populations within each region (GoA-LME = LnL (restricted) = -2171.298; LnL(full) = -1891.803; LRT = 558.978, df = 49, *P <* 0.00001; EBS-LME LnL (restricted) = -inf; LnL(full) = -1153.68; LRT = inf, df = 4, *P <* 0.00001). Maximum likelihood estimates based on Markov Chain Monte Carlo simulations suggest that the direction of gene flow among populations on the Pacific coast is predominantly westward (Fig 6, S4 Table). However, pairwise comparisons between some nearest-neighbor populations (S4 Table) showed exceptions, particularly the predominantly eastward gene flow between North Pacific populations KIL and UNGA, AKSI and WB, and between AKSI and WB (Fig 6). Similar analyses for Bering Sea populations suggested most comparisons involved cyclonic polarity, as movement was eastward, then northward (S3 Table, Fig 6).

## Discussion

### How many species of Zostera were observed in Alaska?

This study represents the first characterization of macrogeographic population genetic parameters among undisturbed meadows of *Z*. *marina* in the extreme high latitude portion of its Northeast Pacific distribution. As such, it provides a foundational assessment of genetic diversity of eelgrass in the region. Fundamental to assessing genetic structure of *Z*. *marina* populations, however, is ensuring the samples representing locales within the Northeast Pacific are indeed *Z*. *marina*, particularly given the proposal that Alaska hosts more than one species of *Zostera* [14]. The ability to identify units of significant evolutionary divergence is a critical first step for assessing regional biodiversity, particularly within the context of management and conservation [98], for clarifying the role of phenotypic plasticity in adaptation [99], and robust interpretation of population genetics data by ensuring that solely within-species analyses are conducted. This is particularly pertinent to *Z*. *marina*, which demonstrates large phenotypic plasticity [16,100], contributing to misidentification of specimens. Morphological differences within and between *Zostera* species include variation in leaf morphology, particularly leaf width. As width of leaves are influenced by both genetic and environmental factors [63], they easily complicate taxonomic assessments [27,51].

The use of sequence data from the nuclear ITS and chloroplast *mat*K genes has clarified species relationships within *Zostera* [16,51]. For example, large genetic differences, but only subtle morphological characteristics, differentiate *Z*. *marina* and *Z*. *japonica* [16], which both occur along the north Pacific coast of North America south of Alaska [17] and possibly in the Alexander Archipelago of Alaska (i.e. UAM Herb: 248738–41). To date, we have failed to uncover *Z*. *japonica* nuclear ITS and chloroplast *mat*K sequences among the samples from Nakwasina and Crab Bay in the Alexander Archipelago (this study and [15], or in 20 other samples assayed from other coastal Alexander Archipelago locales (GenBank accessions: KT272031-50). The nonindigenous invasive, *Z*. *japonica*, has been found in Humboldt Bay, California, and Boundary Bay, British Columbia [63], suggesting the species is spreading both southward and northward from Puget Sound, where it was thought to have been initially introduced [15].

Further, based on variation in leaf morphology among *Zostera* specimens collected in Alaska, Elven [14] considers *Z*. *marina* to occur in single-species meadows in southwest and south Alaska, but admix in northwestern locales with another species, *Z*. *angustifolia*. Elven [14] suggests that *Z*. *marina* and *Z*. *angustifolia* are separable based on quantitative and qualitative vegetative features (width of leaves, number of veins in leaves, degree of branching on stem, size of stigma, and size of fruit [101–104]), citing as supporting evidence of species distinction the observation that mixed stands of both *Z*. *marina* and *Z*. *angustifolia* occur frequently and without observed intermediates. However, the taxonomic status of *Z*. *angustifolia* is disputed; while some authors consider *Z*. *angustifolia* to comprise a subspecies [101] or a distinct species [105,106], others consider it an ecotype of *Z*. *marina* [107–109]. Recently, Becheler et al. [63] demonstrated genetically that ‘*Z*. *angustifolia*’ found along the coast of Brittany represented an ecotype of *Z*. *marina* (*Z*. *marina* v. *angustifolia*) that occurs in areas with strong fluctuations in environmental conditions such as salinity, temperature, pH, and oxygen concentration at low tide. Similar to Becheler et al. [63], who was unable to distinguish ‘*Z*. *angustifolia*’ from *Z*. *marina* in Brittany, we observed no genetic signatures in samples collected in Alaska that were consistent with the presence a ‘*Z*. *angustifolia*’ taxon that differed from *Z*. *marina*

The nuclear ITS and chloroplast *mat*K sequences assayed from among our Alaskan samples were identical to previous assays of *Z*. *marina* [16]. Unfortunately, no ITS or *mat*K sequences attributed to the disputed taxon *Z*. *angustifolia* are archived in public databases, so inference using data from those loci was not possible. Nevertheless, we failed to find any differences in ITS or *mat*K sequences between vouchered specimens determined to be *Z*. *marina*, and the specimen annotated in UAM as *Z*. *angustifolia* (UAM Herb:40475) or among individuals representing Alaskan populations within the distributional ranges proposed by Elven [14] for *Z*. *angustifolia* and *Z*. *marina*. In future situations in which leaf size is a determining factor in *Zostera* species determination, we recommend the use of molecular data in tandem with morphological data to ensure accurate taxonomic classification of specimens; this is particularly important in cases that expand the distribution of a species.

Microsatellite markers are of limited use for determining species [110–112], due to their particular mode and rate of mutation and attendant size homoplasy, high level of within-species variation, and increased likelihood of null alleles in species-level comparisons. Thus, we relied solely on sequence information from the nuclear and chloroplast genome to uncover species-level diversity. However, in some cases, fragment data from microsatellite loci can be used to support phylogenetic inference drawn from sequence data, and can uncover potential cryptic species by revealing genetic characteristics such as diagnostic alleles [113], or linkage disequilibrium and deviations from HWE, expected when analyzing assemblages comprising individuals from more than one species, or hybrid zones [114,115].

We found no evidence from the microsatellite analyses suggesting more than one species occurred among our samples; we found no global or population-wide signature of linkage disequilibrium or deviations from HWE in microsatellite loci, expected if our population samples included individuals belonging to different species. Becheler et al. [63] suggested that ‘*Z*. *angustifolia*’ represents an ecotype that manifests ‘above a perturbation threshold’ (p. 2402) in extreme expression of phenotypic plasticity and facilitates survival of *Z*. *marina* in stressful and fluctuating environments. Although many (if not all) of the locales assayed in our study arguably fall under the category of “stressful and fluctuating,” our research was not designed to specifically test their hypothesis. Nevertheless, our results are in accord with Becheler et al. [63] in that we failed to uncover deep (species-level) divergence among assayed samples that would be expected if two species (*Z*. *marina* and *Z*. *angustifolia*) were sampled from the same locale. Thus, it is possible that specimens recently attributed to *Z*. *angustifolia* in Alaska are, as in Brittany, an ecotype of *Z*. *marina*, rather than a discrete species. Combined, our data suggest that, currently, a single species of *Zostera*, *Z*. *marina*, occurs in Alaska, and as elsewhere is characterized by wide phenotypic plasticity even within the same sampling locale (i.e. Wide Bay). Certainly, however, given the importance of comprehensive sampling to uncover the presence of rare, cryptic species or recent invaders, further sampling and ongoing monitoring is warranted.

#### Genetic differentiation and structure

Significant within-species structuring was evident among almost all locales studied, as demonstrated by the analyses of variance in allele frequencies using traditional F-statistics as well as Bayesian clustering analyses, with at least nine clusters among the 12 locales analyzed. Further, this divergence is in general not extremely recent [75,116]; R_ST_ (ρ) values are larger than overall F_*ST*_ (θ) values, and for 54% of all pairwise comparisons, ρ values are larger, and typically substantially so, than θ values. This pattern is repeated within regions: ρ_*ST*_ values were greater than θ_*ST*_ values in 11 of 21 pairwise population comparisons within the GoA-LME, and in eight of the 10 pairwise population comparisons within the EBS-LME. Interestingly however, θ_*ST*_ exceeded ρ_*ST*_ values in a small majority of between-region comparisons (53%), involving mostly populations occurring near the tip of the Alaska Peninsula.

Studies of eelgrass populations in the northwestern North Pacific [21], along the southern Pacific coast of Baja California and the Gulf of California [38], certain sites in Puget Sound, Washington [58] and San Francisco Bay, California [117], and in Atlantic ecosystems [37], show that eelgrass in both the Pacific and Atlantic display pronounced population structuring, sometimes even among sites within the same lagoon. Given the perennial habit of *Z*. *marina* along most of the north Pacific coast of North America, and the observation that movement of dislodged vegetative material is the main adaptation the fruits have for dispersal [17,107], a finding of significant structuring is not surprising. The apparent low propensity for dispersal, corroborated by repeated findings of pronounced population structuring, has implications when assessing the impact of catastrophic anthropogenic or natural perturbations on local *Z*. *marina* populations and the success of restoration programs. However, most of these studies have been conducted in areas already heavily impacted by anthropogenic activity, which can increase population isolation via fragmentation [118], complicating interpretation of results within the context of species-specific evolutionary strategies across different marine ecosystems.

Although we observed significant genetic structure among the high latitude populations in both the EBS- and GoA-LMEs, regional (inter-LME) differentiation was only significant if we forced KIL, located along the Pacific coast of the western Alaska Peninsula and therefore within the GoA-LME, to group with populations in the EBS-LME (Models C, D, and H; S2 Table). As in other regions along the Pacific coast of North America [38], we found that the Alaska Peninsula likely limits, to some degree, gene flow among eelgrass populations between the two LMEs, since among AMOVA models tested, the highest significant ***θ*_*p*_** value was observed when populations were generally partitioned into three regional groupings, corresponding to 1) populations in the Bering Sea (but also including KIL); 2) populations along the southern portion of the Alaska Peninsula; and 3) populations farther east, along the Gulf of Alaska and the northernmost Pacific coast (Alexander Archipelago). However, given the close relationship of KIL with Bering Sea populations, this boundary appears to be semipermeable for eelgrass populations around the tip of the Alaska Peninsula, where currents flow from the GoA-LME to the EBS-LME. Nevertheless, under the SMM, these partitions were not identified as significant, suggesting observed differentiation among these three regions is an evolutionarily shallow phenomenon, with connectivity still maintained by low levels of gene flow [75].

The combination of unexpected net eastward gene flow among certain populations occupying the Gulf of Alaska (KIL, WB, UNGA and AKSI; Fig 5) and lack of a significant isolation-by-distance signal suggests that net current movement may play a less than central role in the dispersal of eelgrass among certain Alaskan populations. The variation in strength of flow and current polarity seems insufficient to explain the large amount of genetic connectivity between the EBS- and GoA LMEs seen among SCC, IZL, and KIL, and the distinctiveness of WB, which as a clearly isolated lagoon is relatively protected from net ocean current movement (and therefore likely gene flow) that connects other GoA-LME eelgrass populations. Seasonal divergences in net current polarity along the Pacific Coast of the Alaska Peninsula may account for some the observed disparities; in summer, surface currents along the Pacific coast of Alaska develop into weak, closed, anticyclonic patterns [119,120], at approximately 5.1–25.5 cm·s^-1^, displacing the winter net northward flowing Alaska Current to the west [121]. However, summer flows are weak and the strength of the circulation varies annually [119]. In contrast, net surface flows along the Alaskan coast in the Bering Sea are predominantly to the north and northwest, with only weak sporadic current flowing southward along the west coast of the Bering Strait [122]. Strength of flow through the strait apparently varies by a factor of five within the space of weeks. Current velocities along the northern coast of the Alaska Peninsula, where the densest meadows of eelgrass in North America occur [12,13], range from 5–34 cm·s^-1^ in the channels, and from 0–18 cm·s^-1^ in the meadows [123].

In *Z*. *marina* populations in Europe, genetic distance increases with increasing geographic distance [57], and genetic characteristics of populations in the GoC-LME in Mexico has been explained, at least partially, relative to currents and oceanographic mixing patterns [38]. Gene flow and population dynamics for marine organisms, such as seagrasses, can certainly be influenced by the distance between populations, currents and oceanographic mixing patterns [124], and as discussed may account for the genetic isolation of the locally isolated sampling locale in Wide Bay. However, gene flow may also be facilitated by birds, fish or humans [125–129]. Izembek Lagoon is a fall staging site for large numbers of migrating birds, including over 90% of the Pacific population of brant (*Branta bernicla nigricans*), which feed exclusively on eelgrass prior to embarking on long-distance flights to wintering areas in Mexico [130,131], and numerous duck species which are known to feed on seeds of eelgrass in the fall [29,125,132]. Thus, waterfowl may play a major role in the short-distance dispersal of eelgrass in IZL [126,132], and this may also be true for nearby populations along the southern portion of the Alaska Peninsula, particularly WB, which appears to receive gene flow from both the east and the west. Clearly, the relationships among populations occupying habitats along and on the tip of the Alaska Peninsula, whether on the Bering or North Pacific side, are complex, and additional investigation of this region, including investigation of mechanisms of dispersal other than ocean currents, is warranted. As a result, we are presently designing research to address the potential of short-distance dispersal mechanisms on the distribution of genetic variation in eelgrass of Alaska.

### Levels of genetic diversity

Other studies [20,21,37,38,58,117,133] have reported a wide range of genotypic diversity among eelgrass populations, ranging from complete monoclonality (in Europe) to maximal diversity (Mexico). Levels of genetic variation at the microsatellite loci used to study the populations of *Z*. *marina* along the northern Gulf and Bering Sea coasts of Alaska are lower relative to those observed at the same loci in eelgrass populations to the south. For example, average heterozygosity (H_E_) in eelgrass populations in Baja California was approximately 0.6, and mean number of alleles (A) approximately 5.5 [38] and those values are similar to values reported from populations of *Z*. *marina* in the north Atlantic and Europe [57] and Japan [21]. However, average heterozygosity in Alaskan populations at the same loci was approximately 0.4, and mean number of alleles was 4.1, and we observed a strong inverse correlation between genetic diversity with increasing latitude. Sampling regimes or sample selection used in this study were comparable to those employed by Muñiz-Salazar et al. [38] and Wyllie-Echeverria et al. [58]; thus we do not attribute the disparity in levels of genetic diversity to differences in sampling, except for KS for which samples may have been collected at < 20m intervals. Coyer et al. [96] posit that southern populations along the Pacific coast, particularly in the California Bight, show increased diversity due to introgression between *Z*. *marina* and another proposed species, *Z*. *pacifica*. The genetic criterion used by Coyer et al. [96] to distinguish between these two species was based on their failure to amplify a single microsatellite locus, CT20, in the proposed *Z*. *pacifica* (and not based on any diagnostic differences in sequences from the nuclear and chloroplast genome). The potential presence of another species in that region cannot account for our observation of increased genetic diversity at lower latitudes; all genotypes from all individuals used in our comparative analyses, including those from California (MON), amplified a product at CT20. As well, we observed no global signal of linkage disequilibrium, expected in cases of recent introgression [63], within any of the lower latitude populations used in our comparisons (i.e. Baja California populations [38]; MON, http://dx.doi.org/10.5066/F7GQ6VTK).

The observed pattern of reduced genetic variation in Alaskan eelgrass populations is consistent with observations in other seagrass species, such *Posidonia oceanica* L., an endemic Mediterranean seagrass [134]. Like *P*. *oceanica*, *Z*. *marina* is characterized by a clonal reproduction habit, reproducing in two ways: sexually by means of flowers (albeit functionally hermaphroditic rather than moneoecius [135]), and asexually via extensive vegetative propagation [136]. While both perennial and annual meadows of *Z*. *marina* inhabit coastal regions along the southernmost portion of the species’ range and in disturbed meadows along the mid portion of the Pacific coast [117], in Alaska, eelgrass is characterized by perennial life history, reproducing sexually during the short summer months, but also growing actively through clonal reproduction. Thus, it is not surprising that the majority of allelic variation characterizing eelgrass meadows in Alaska was distributed among, and not within, populations [137].

We have already pointed out that different populations of *Z*. *marina* demonstrate a remarkable level of morphological and physiological variability, and leaf width and length often correlates with habitat [17,108,138,139]. This is evident with our finding that a specimen determined to be *Z*. *angustifolia* (UAM Herb: 40475) based on morphological characters (discussed earlier) nevertheless possesses identical chloroplast and nuclear sequences with *Z*. *marina* from elsewhere in the North Pacific, suggesting the specimen is actually *Z*. *marina*. Here, we found no evidence of large genetic differences among individuals taken from the same site, but at different locations within the tidal zone. Results from our population genetics assessment–i.e. lower to moderate levels of genetic diversity, and relatively higher levels of clonality in northern when compared to southern populations (e.g., Baja California and Sonora [38])–support the hypothesis of a life history in which perennial plants reproduce by vegetative propagation, but in which vegetative reproduction is augmented by seasonal flowering; the percentage of flowering to non-flowering *Z*. *marina* shoots is lower in Alaskan populations [44]. Thus sexual reproduction may play a less important role in the life cycle of these northern meadows, with vegetative reproduction more dominant, resulting in decreased levels of genetic variation, than for eelgrass meadows in the southern portion of the species’ range where higher levels of genetic variation at the loci examined characterize all populations [38,39]. Even in southern meadows characterized by perennially and low incidence of flowering, such as San Quintin [140], reproductive shoots appear earlier and persist longer, relative to northern populations, and reproductive shoots represent up to 10% of the total shoots in eelgrass meadows. Nevertheless, seed densities of approximately 4,000 seeds m^-2^ reported from San Quintin [140] are lower than seed densities reported from more northern lagoons, estimated at between 6,862 seeds m^-2^ at Hole in the Wall (outer coast of Washington State) to from 700 to 15,000 m^-2^ at IZL [44]. Thus the decreased levels of genetic variation and increased clonality observed in Alaskan populations relative to southern populations cannot be fully explained in terms of reproductive strategies alone. Also consistent with low levels of genetic variability of the north is that Alaskan populations represent the leading edge of populations expanding out of southern refugia subsequent to climate amelioration following the last Pleistocene glacial period [52].

### Pacific origin of extant *Z*. *marina* populations?

The evolutionary origins of seagrasses remain obscure, but given the low number of seagrass species, Green and Short [141] suggest that the group might be of recent evolutionary origin (although see den Hartog [107]). Biogeographic evidence points to the tropical Indo-Pacific as the center of origin for seagrasses, but the northern Pacific (particularly the northwestern Pacific) as the origin for temperate (*Phyllospadix* and *Zostera*) species [5,142]. Although Kato et al. [62] placed the divergence of the four *Zostera* species to between three and six million years ago (mya), based on an ITS clock for algae and other green plants [143], Olsen et al. [37] estimate that *Z*. *marina* originated in the Pacific between eight and 20 mya, during the Neogene epoch. Olsen et al. [37] screened North Atlantic and a small number of northeastern Pacific (Padilla Bay, WA; and Auke Bay, southeastern Alaska) populations of *Z*. *marina* and observed higher levels of genetic diversity within the Pacific populations. Although eastern Pacific and western Atlantic populations appear to be connected, western Pacific and eastern Atlantic populations of eelgrass appear less so, prompting Olsen et al. [37] to argue in favor of an eastern Pacific origin for western Atlantic *Z*. *marina* via a trans-Arctic connection, as hypothesized for a number of marine taxa (see [144]). However, as pointed out by Olsen et al. [37], glacial-interglacial cycling of the late Quaternary likely facilitated extirpation-recolonization cycles (i.e. Provan and Bennett’s [145] expansion-contraction model of Pleistocene biogeography) that may have greatly impacted the distribution of genetic variation in contemporary high-latitude eelgrass populations.

The Pleistocene was characterized by worldwide climate change associated with glacial cycles, and while the fossil record reflects these changes for terrestrial species [146–148], the fossil record has been less informative for arctic and subarctic marine species [149]. Expansion and retraction cycles of ice sheets in the northern continents dramatically affected the distribution of terrestrial floral and faunal species there (see [52,150] for reviews), with many species shifting ranges, often to more southerly and warmer refugia, when large expanses of the boreal zone were covered with ice sheets and permafrost. Under the expansion-contraction model and given Hewitt’s ‘southern richness, northern purity’ model [52], repeated range contractions and northern extinctions of thermophilic species throughout the Pleistocene, coupled with survival of populations only in unglaciated southern portion of species’ ranges, would result in a phylogeographic pattern in which northern populations would harbor less genetic diversity than southern populations. Following climate amelioration, these populations expanded northward as they tracked changing environments. Under this model, a pattern of low genetic diversity coupled with a small number of alleles or haplotypes over large geographic areas in high latitudes would signal a recent (post-Pleistocene) range expansion from a more southern refuge.

Although marine deposits from interglacial periods are fossil-rich and easily accessible, glacial fossil records from arctic and subarctic marine species are largely inaccessible due to sea level rise during the Holocene [149]. As a result, molecular approaches to the study of genetic variation in marine species have significantly impacted our understanding of the consequences of past episodes of global climate change on marine species and populations in this region. Molecular surveys of marine organisms across high latitudes have yielded a number of models relating patterns of genetic differentiation to potential historical isolating events, particularly Quaternary glaciation, including expansion of species from northern periglacial refugia along the European continent (reviewed in [151]) and the northwest [152] and northeast [144,153,154] Pacific Ocean. Along the coast of Alaska, advancing ice sheets and lowered sea levels coupled with expanded shoreline habitat during the LGM is thought to have fractured contiguous populations of certain cold-adapted intertidal species, such as brown algae *Fucus distichus* [144], or cryptic warm-adapted species held in refugia near or south of Washington State [155–157], generating a number of small, relatively isolated refugial populations. However, a pattern of significant latitudinal clines, with low genetic diversity at relatively high northern latitudes, consistent with Hewitt [52] and Provan and Bennett [145], has also been observed in a number of marine taxa (*Balanophyllia elegans* [158]; *Nucella ostrina* [154]), including possibly eelgrass in the North Pacific coast of North America.

Marko [154] posits that low levels of genetic variation in high latitude marine species–suggestive of recent colonization from southern refugia–is more common among intertidal species that live relatively high on the shore, as exposure time to cold stress in air would be longer than for species that live lower in the shore. Our analyses show that at least two measures of genetic diversity at microsatellite loci is inversely correlated with latitude in eelgrass; this and the complete lack of variation in both the *matK* and ITS genes in both high latitude LMEs (this study and [15] fails to support a northern high latitude refugium hypothesis. Given their greater genetic diversity, populations in more southern locales along the Pacific coast of North America may represent a late Pleistocene refugial origin of contemporary northeast Pacific eelgrass populations, but the *mat*K variant characterizing the north was not observed in populations south of Humboldt Bay, or reported elsewhere. The distribution of *mat*K haplotypes along the temperate Pacific coast is suggestive of one or two broad biogeographic breaks (sharp intraspecific genetic discontinuities). The first is suggested by the distribution of *mat*K haplotypes that corresponds to a break, well-supported based on distribution of other marine species, near Cape Mendocino in northern California [159]. The second break is suggested by the distribution of ITS haplotypes at or near Point Conception and the Los Angeles region in southern California, hypothesized for eelgrass based on analyses of microsatellite loci in populations along the Baja California peninsula [38], as well as a number of other marine species [160]. Clearly, additional sampling is required to test the conformation of the distribution of genetic differentiation in *Z*. *marina* to these biogeographic breaks.

We suggest that extant populations in the Northeast Pacific likely reflect a leading edge northward Holocene expansion of populations held in temperate LGM refugial populations (either the Northwest Pacific, or Northeast Pacific populations south of the ice sheets), that, to date, appear to have reached only as far north as 66.52° N (Cowpack Inlet, N. arm of Shishmarf Inlet, Seward Peninsula, UAM 40475), the northernmost documented occurrence of the species in the Northeast Pacific. Olsen et al. [37] cite the long-distance dispersal of bathroom toy ducks from the northeastern North Pacific to Iceland [161] as evidence of an ongoing trans-Arctic oceanic pathway for long-distance colonization of North Atlantic by Northeast Pacific eelgrass populations. However, under that model, we would expect the most common northwest Pacific (Japan) *mat*K haplotype (*mat*K2), which is fixed in Alaskan populations, to be present in the North Atlantic. To date, variant *mat*K2 (Zmm1 [AB125354] of Kato et al. [62]) has not been reported outside the north Pacific. However, *mat*K haplotypes found in California and Mexico (*mat*K4), but not in Alaska, are identical to those in samples collected from Atlantic and, at lower frequency, in Japanese waters [62,96]. Thus, our results lend little support for a trans-Arctic oceanic pathway from the northeast North Pacific to the Atlantic, at least during the Holocene. We cannot yet rule out long-distance Pacific to Atlantic dispersal of eelgrass by migrating ducks, geese, or other waterbirds.

## Supporting Information

S1 Fig Distribution of two ITS alleles and five *mat*K alleles. *Z*. *marina* samples collected from locales in the EBS-, GoA-, CC- and GoC-LMEs along the Pacific Coast of North America, and Japan. GenBank accession numbers containing sequence data from a representative of each haplotype are provided, selected from GenBank accession numbers KU704921-KU705084 (*mat*K), and KU704817-KU704920 (ITS). Asterisks (*) indicate locales for which sequence data from Talbot et al. [16] are included (GenBank accessions: KU562104-KU562118; KU596406-KU596418). NB = Notuke Bay, Hokkaido, Japan; LF = Lake Furen, Hokkaido, Japan; MOR = Morro Bay, California; EPB = Estero Punto Banda; BSQ = Bahia San Quintin; LOL = Laguna Ojo de Liebre; SI = San Ignacio; BC = Bahia Concepcion; IST = Canal del Infiernillo, SON = Punta Chueca, Sonora.
(TIF)

S2 Fig Plot of results of Bayesian clustering analysis using BAPS5.1 [81] under admixture analysis. (A) 9 genetic clusters estimated from the microsatellite data under admixture models for K = 1–20, and clusters when analyses were constrained to partition clusters when (B) K = 2, (C) K = 3, and (D) K = 4. Individual samples are represented by a single vertical line along the x-axis, according to sampling locale, which are delineated from other sampling locales by a black vertical line.
(TIF)

S3 Fig Optimal clustering and membership probability by population. (A) Comparison of sampled populations to the optimal number of clusters in these data. Inferred by k-means (k = 15). (B) Plot of membership probability of each individual to each sampled population using DAPC. Implemented in *adegenet* (k = 12) [79].
(TIF)

S1 Methods Detailed methods of laboratory and data analyses. (DOCX)

S1 Table Pairwise *θ*_*ST*_ (below the diagonal) and ρ_*ST*_ (above the diagonal) values among populations occupying the Bering Sea and North Pacific coasts of Alaska. (DOCX)

S2 Table Analyses of molecular variance (AMOVA) for hypothesized groupings, based on fragment data from ten microsatellite loci. (DOCX)

S3 Table Pairwise estimates of directional gene flow (*N*_*e*_*m*) and Θ for each population, using 10 microsatellite loci, among EBS-LME populations. (DOCX)

S4 Table Pairwise estimates of directional gene flow (*N*_*e*_*m*) and Θ for each population, using 10 microsatellite loci, among GoA-LME populations. (DOCX)
