# Supplementary figures and images for: The Structure of Genetic Diversity in Eelgrass (Zostera marina L.) along the North Pacific and Bering Sea Coasts of Alaska
Source: PLoS One. 2016 Apr 22;11(4):e0152701. doi: 10.1371/journal.pone.0152701 (PMC4841600; doi:10.1371/journal.pone.0152701)

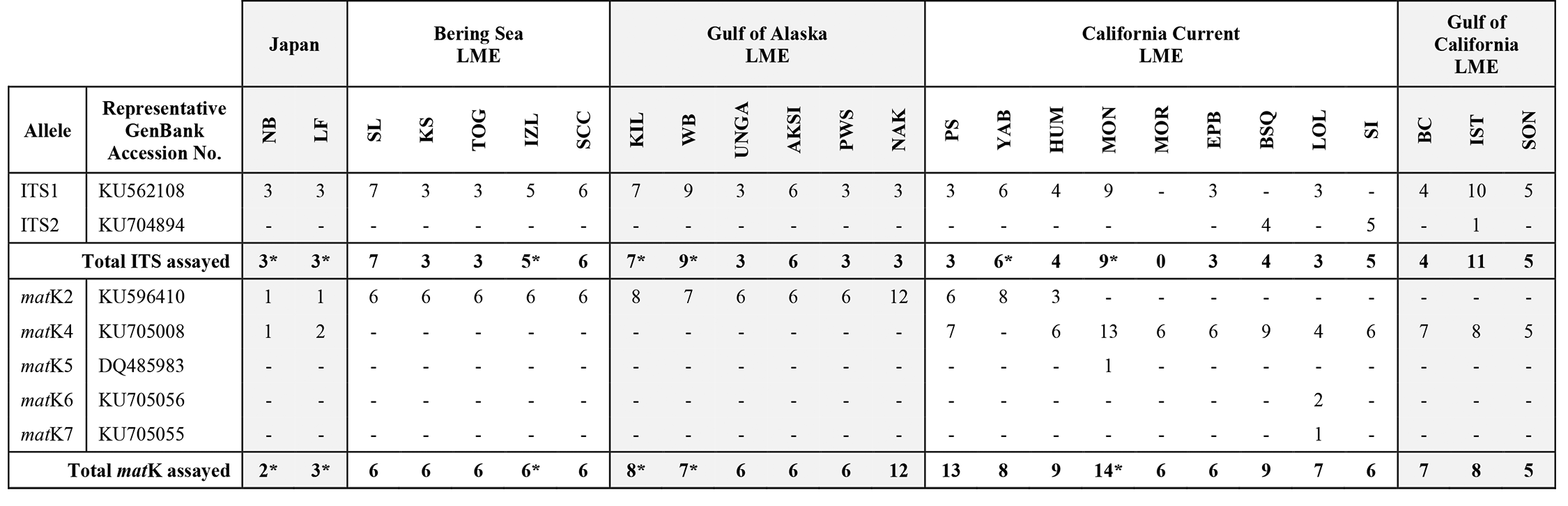

Supplement: S1 Fig — Z. marina samples collected from locales in the EBS-, GoA-, CC- and GoC-LMEs along the Pacific Coast of North America, and Japan. GenBank accession numbers containing sequence data from a representative of each haplotype are provided, selected from GenBank accession numbers KU704921-KU705084 (matK), and KU704817-KU704920 (ITS). Asterisks (*) indicate locales for which sequence data from Talbot et al. [16] are included (GenBank accessions: KU562104-KU562118; KU596406-KU596418). NB = Notuke Bay, Hokkaido, Japan; LF = Lake Furen, Hokkaido, Japan; MOR = Morro Bay, California; EPB = Estero Punto Banda; BSQ = Bahia San Quintin; LOL = Laguna Ojo de Liebre; SI = San Ignacio; BC = Bahia Concepcion; IST = Canal del Infiernillo, SON = Punta Chueca, Sonora. (TIF) [file pone.0152701.s001.tif]

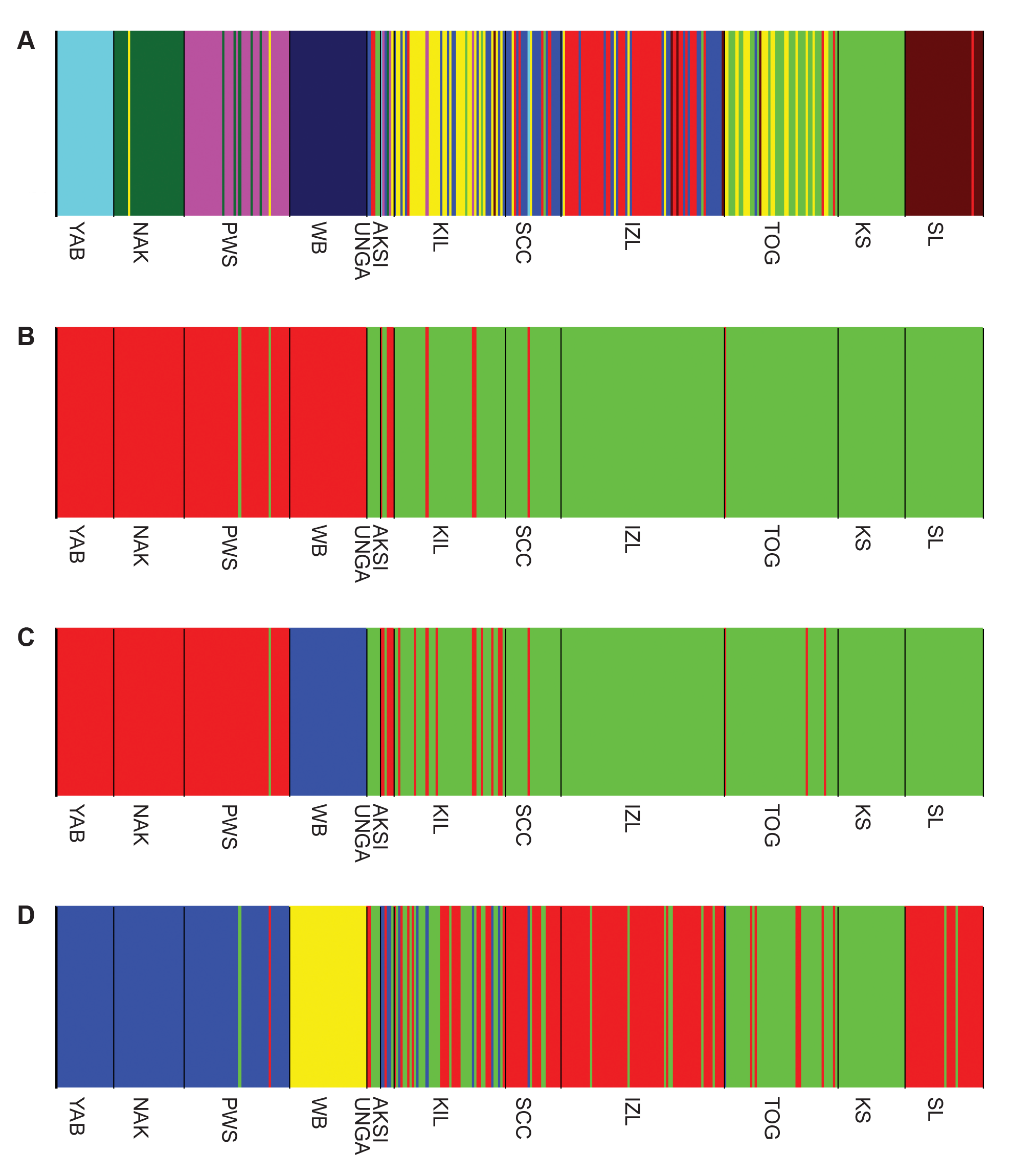

Supplement: S2 Fig — (A) 9 genetic clusters estimated from the microsatellite data under admixture models for K = 1–20, and clusters when analyses were constrained to partition clusters when (B) K = 2, (C) K = 3, and (D) K = 4. Individual samples are represented by a single vertical line along the x-axis, according to sampling locale, which are delineated from other sampling locales by a black vertical line. (TIF) [file pone.0152701.s002.tif]

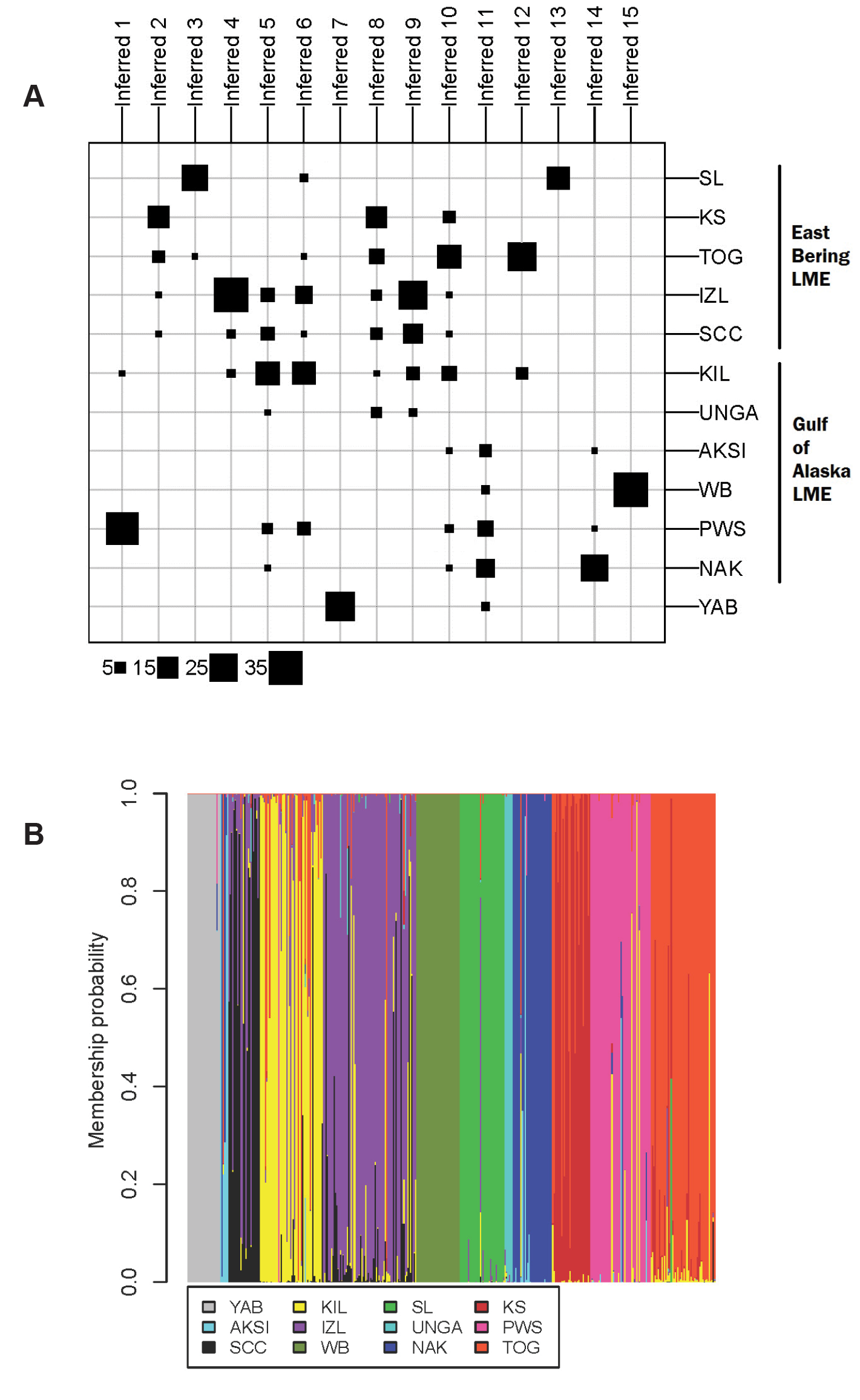

Supplement: S3 Fig — (A) Comparison of sampled populations to the optimal number of clusters in these data. Inferred by k-means (k = 15). (B) Plot of membership probability of each individual to each sampled population using DAPC. Implemented in adegenet (k = 12) [79]. (TIF) [file pone.0152701.s003.tif]
