# Supplementary material for: The Structure of Genetic Diversity in Eelgrass (Zostera marina L.) along the North Pacific and Bering Sea Coasts of Alaska
Source: PLoS One. 2016 Apr 22;11(4):e0152701. doi: 10.1371/journal.pone.0152701 (PMC4841600; doi:10.1371/journal.pone.0152701)
Supplement: S1 Methods — (DOCX) [file pone.0152701.s004.docx]

**S1 Methods. Detailed methods of laboratory and data analyses**

**Laboratory analyses**

Genomic DNA was extracted from approximately 0.02-0.04 g dry weight of leaf tissue and quantified using methods outlined in Muñiz-Salazar et al. [1]. We verified species by gathering nucleotide sequence information from approximately 348 basepairs (bp) of the *Zostera* chloroplast maturase K (*mat*K) gene and 520bp of the 5.8S rRNA gene and associated internal transcribed spacers, ITS-1 and ITS-2 (ITS) from 3-12 individuals in each population, and additional vouchered samples archived at UAM (see below). Sequences were obtained from product generated via the polymerase chain reaction, using primers and processing methods outlined in Talbot et al. [2]. For comparative purposes, we also obtained sequence data from *mat*K and ITS from 2-12 individuals per population (except Morro Bay for ITS) in the other two Pacific Coast LMEs of North America: 1) the California Current LME (CC-LME), including Puget Sound, Washington (PS), Monterey (MON), Humboldt (HUM), and Morro bays, California, and four lagoons along the coast of Baja California Peninsula (Estero Punto Banda, Bahia San Quintin, Laguna Ojo de Liebre, Laguna San Ingnacio [1], and three lagoons in the 2) Gulf of California LME (GoC-LME: Bahia Concepcion, Punta Chueca, and Isla Tiburon [1]). All *mat*K and ITS data were compared with data from prior research [2] and sequences accessioned in GenBank (KU704817-KU705084).

DNA samples from Alaskan locales were genotyped at 10 polymorphic microsatellite loci (Zosmar CT-3, CT-12, CT-17, CT-19, CT-20, GA-1, GA-2, GA-3, GA-4, GA-5; [3,4]), following procedures outlined elsewhere [1,5]. For comparative purposes, genotypes generated from YAB (see Muñiz-Salazar et al. [1]) were also included in analyses.

**Data analyses**

All *matK* and ITS sequences were compared using the BLASTN algorithm [6] to data from different *Zostera* species deposited on NCBI (see [2,7,8]). As well, sequences were compared to homologous information from both loci obtained from accessioned specimens used by Talbot et al. [2] to represent North Pacific *Z. marina*: UAM Herb:43460, collected from Wide Bay, Alaska Peninsula (GoA-LME); and UAM Herb:40475, collected from Cowpack Lagoon on the Seward Peninsula (EBS-LME); we note that UAM Herb:40475 was annotated and ascribed in 2006 to *Z. angustifolia*. Although Elven [9] has proposed that *Z. angustifolia* is co-distributed with *Z. marina* in Alaska, prior research [2] failed to uncover any variability in ITS or *mat*K sequence in *Zostera* samples collected from six widely-separated locales in Alaska (NAK in southeast Alaska, WB in the middle Alaska Peninsula, IZL and KIL lagoons at the tip of the Alaska Peninsula and Cowpack Lagoon on the Seward Peninsula), suggesting a single species of *Zostera*, presumably *Z. marina*, occurs there. Unfortunately, no sequence data for ITS or *mat*K attributed to *Z. angustifolia* are archived in GenBank. Nevertheless, we assumed that if two different *Zostera* species occupied habitats within the two high latitude LMEs, we would observe 1) diagnostic differences between UAM Herb: 40475 (cf. *Z. angustifolia*) and UAM Herb: 43460 (*Z. marina*) at the ITS and *mat*K genes, and 2) among sequences assayed from individuals representing our target populations. Further, 3) if sampled ‘populations’ actually comprised individuals from different species, we should observe significant linkage disequilibrium (and likely deviations from Hardy Weinberg proportions) across most or all microsatellite loci collected across individuals sampled from within the mixed ‘population’ [10].

**Analyses of sequence data**

*Mat*K and ITS sequences were collapsed into haplotypes using DNACollapser [11]. An unrooted phylogenetic network was constructed for *mat*K data using NETWORK Version 4.612 (Fluxus Technology Ltd., fluxus-engineering.com; [12]). Homologous sequence from three other species of *Zostera* (*Z. asiatica*: AB125360; *Z. japonica*: AB125361; and *Z. caulescens*: AB125358; [8]) were included for comparison.

**Clonality and genetic diversity**

We assessed clonality by examining match statistics for multilocus genotypes among samples, using Microsatellite Toolkit [13] to identify 0MM, 1MM and 2MM matches [14] and verify raw scores, and GenClone ver. 1.0 [15] to estimate levels of clonality. Here, we define a sample that has a unique ten-locus genotype as a multilocus genotype that is treated as equivalent to a genetically unique individual, or genet. Clonality is presumed between two or more samples when each are identical at all of the loci compared, when the number of loci compared gives sufficient power (e.g., P_IDsib_ < 0.01, derived from analyses of presumed genets only; see results). Genotypic richness (R), a measure of clonality within the population, was also evaluated using GenClone, which employs the method of Dorken and Eckert [16]. Populations with a value of 1.0 for R have 0.0% clonality; lower values of R indicate higher levels of clonality. Following assessment of R, all further analyses were conducted on genets only; that is, when multiple samples share the same multi-locus genotype, only one sample is used to represent the genet. The pruned dataset was used to test the power to detect individuals by calculating the probability of observing identical multilocus genotypes between two individuals sampled from a population (P_ID_ and P_IDsib_; [17]), using the program GIMLET v. 1.3.2 [18].

For genets only, we calculated allele number and heterozygosities (observed [H_O_] and expected [H_E_]) for each locus across all populations using GDA 1.0 [19], and percent polymorphism (P) using GenAlex 6.0 [20]. Allelic richness (AR) per locus and population [21,22] was calculated using FSTAT 2.9.3 [23]. The global test for deviation from Hardy-Weinberg equilibrium (HWE) was performed using a Markov chain algorithm developed by Guo & Thompson [24], and linkage disequilibrium between all pairs of loci was estimated using GENEPOP’007 [25,26]. In all cases of multiple tests, significance levels were adjusted using sequential Bonferroni corrections [27].

**Population structure**

Levels of population structuring were assessed using two standard indices of population differentiation: analysis of interpopulational variance using frequency-based F-statistics (the F*_ST_* [28]) and size-based analogs (the R*_ST_* [29,30]), and allelic goodness-of-fit. F*_ST_* estimators (and analogs) and allelic goodness-of-fit tests are more powerful than genotypic goodness-of-fit tests, and, when sample sizes are unequal, allelic goodness-of-fit tests are the most powerful [31].

A global multilocus estimate of F*_ST_* (***θ*** [28]) was obtained using the program FSTAT (Ver. 2.9.3 [23]). Estimates of interpopulational variance (***θ****_ST_*) were derived using the program ARLEQUIN 3.1 [32]. Significance of ***θ*** values were based on random permutation tests (n =1000), whereby alleles were randomly permuted between the two populations. We also calculated **ρ**, a global multilocus estimate analog of F*_ST_* [29] which assumes a stepwise mutation model (SMM [33]) that is derived from variances in mean allele size and frequency in relation to sample size and seen as a more conservative distance measure relative to F*_ST_* [30]. Statistical significance of **ρ** was tested in the same manner as ***θ***; for both tests, p-values were adjusted using Bonferroni corrections. Population differentiation based on the distributions of alleles and genotypes across populations was examined using a log-likelihood (G) based exact test [31], implemented by GENEPOP’007 [26] and judging the significance using Fisher’s exact test method and/or by applying sequential Bonferroni procedures.

We also applied a Bayesian clustering approach to further investigate the pattern of population structuring. Unlike averaging methods such as traditional F*_ST_* methods, Bayesian methods combine information from several loci into a single probability model, and are able to detect hidden substructuring through pooling of combinations of the sampled populations (e.g., [34]) or though pooling sets of individuals independently of the actual sample structure (e.g., [35]). Data were analyzed using BAPS5.1 [34] to detect the occurrence of population structure, without a priori knowledge of putative populations, under two situations: situations under which i) the number of clusters was pre-defined (K = 2, corresponding to the two LMEs, and K = 3-4, based on results of the regional analyses of molecular variance; see below); and ii) the number of clusters was not predetermined, but with an upper limit of 20 (more than the sampled locales). The latter analysis was intended to facilitate the identification of more detailed patterns across the geographic distribution. Each Bayesian clustering analysis was repeated 10 times to ensure consistency across runs.

Because traditional as well as Bayesian approaches assume linkage equilibrium and conformation to HWE, and several populations (see results) failed to meet those assumptions, we also conducted a discriminant analysis of principal components (DAPC) as implemented in the R package *adegenet* [36] to investigate genetic structuring and visualize regional and between-population differentiation within the dataset. Optimal cluster number within each region and population was determined using sequential k-means algorithm on principal components and discriminant functions transformed data and compared to original population identification, with probability of membership in each population determined for each sample[37].

**Regional structure**

To test for regional differentiation, we performed hierarchical analyses of molecular variance (AMOVA), testing the hypothesized regional relationship between the GoA-LME (NAK, PWS, UNGA, AKSI, WB, KIL) and the EBS-LME populations (SCC, IZL, KS, TOG, SL), relative to a suite of alternative hypotheses. Genetic variance was quantified at four hierarchical levels for all analyses of variance:1) ***θ****_p_*, between regions (EBS-LME and GoA-LME), 2) ***θ****_s_*, among populations within regions, 3) ***θ****_f_*, among individuals within populations, and 4) ***θ****_F_*, within individuals. Statistical significance of variance measures was assessed via non-parametric permutation [38].

**Population relationships and tests of isolation-by-distance**

We examined genetic relationships among populations by creating a neighbor-joining tree [39], inferred from allelic frequency data using Cavalli-Sforza and Edwards’[40] chord distances (D_CE_ ). Confidence of the tree topology was evaluated by bootstrapping over loci (1000 replicates) using POPULATIONS 1.2.30 [41]. Networks were viewed in TREEVIEW Ver. 1.6.6 [42]. We used tests of isolation-by-distance to evaluate the relative historical roles of gene flow and drift on population structure. Isolation-by-distance tests compare pairwise genetic and geographic distances with those expected under a stepping-stone model of population structure [43]. If gene flow is affected by geographic distance, we would expect adjacent populations to exchange a larger number of migrants. We examined the correlation between logarithm of the geographic distance and Rousset’s genetic distance (F_ST_/ [1–F_ST_]) [44] for populations overall and within each region, separately, using the program IBD 3.0 [45]. Reduced-major-axis regression implemented in the program IBD was used to determine the slope of significant regression for the Pacific coast and Bering Sea graphs.

To determine whether there is an inverse relationship between genetic diversity and latitude, as expected if contemporary populations of eelgrass in the North Pacific colonized from temperate or southern refugia following climate amelioration at the Pleistocene/Holocene boundary, we performed a linear regression analysis fitting expected heterozygosity (H_E_) and standardized allelic richness (AR) data to degrees latitude. An inverse relationship between genetic diversity and latitude is expected if genetic diversity decreases constantly along a latitudinal gradient following an isolation-by-distance pattern. We combined data from this study (YAB, NAK, WB, IZL, TOG, SL only) and comparable data from other populations (Baja California: Bahia Magdalena, Bahia San Quintin, Estero Punto Banda, [1]; California: MON; and Washington: PS, represented by Shallow Bay in [5]). Data for six (CT-3, CT-20, GA-1, GA-2, GA-3, and GA-5) of the 10 loci used in this study were available for all 12 populations, and all H_E_ values were derived from genets, based on their unique MLG. Microsatellite genotype data are accessioned at the USGS Alaska Science Center data repository (http://dx.doi.org/10.5066/F7GQ6VTK).

**Gene flow rates and polarity**

We estimated the magnitude and polarity of gene flow among populations within the two regions using the maximum likelihood approach implemented in MIGRATE 2.0.3 [46,47]. MIGRATE uses a coalescence approach to estimate gene flow rates (N*_m_*) among populations, assuming a constant per-locus mutation rate (μ). This approach is judged to estimate gene flow more accurately than other F_ST_ methods, especially when multiple loci are employed [46]. The program assumes discrete populations and generations, mutation-drift equilibrium, no selective effects, and the SMM for microsatellite markers.

We calculated effective population size as a function of mutation rate (Θ = 4N_e_µ, where N_e_ is the effective population size, µ is the mutation rate, and *m* is the rate of migration into the population), and effective numbers of migrants per generation (4N_e_*m*), using microsatellite data. To test whether net current direction can predict localized direction of gene flow, we performed MIGRATE analyses separately for Bering Sea and Pacific coast populations. Full models, Θ = 4N_e_μ (the composite measure of effective population size and mutation rate) and all pairwise migration parameters were estimated individually from the data and compared to a restricted island model whereby Θ and pairwise migration parameters are constrained to be equal between populations. Comparison between these two models allows us to test for the presence of asymmetry in gene flow and, thus, identify potential sink and source populations within regions.

MIGRATE was performed using maximum likelihood search parameters (10 short chains using 1,000 trees of 20,000 sampled followed by five long chains using 10,000 trees out of 200,000 sampled and five adaptively heated chains (start temperatures: 1, 1.5, 3, 6 and 12; swapping interval = 1). To ensure convergence of parameter estimates, full models were run three times. Restricted models were run once. Competing models were evaluated for goodness-of-fit given the data using a log-likelihood ratio test. The resulting statistic from the log-likelihood ratio test is equal to a χ^2^ distribution, with the degrees of freedom equal to the difference in the number of parameters estimated in the two models [47].

**References**

1. Muñiz-Salazar R, Talbot SL, Sage GK, Ward DH, Cabello-Pasini A. Population genetic structure of annual and perennial populations of *Zostera marina* L. along the Pacific coast of Baja California and the Gulf of California. Mol Ecol. 2005;14: 711–722. doi:10.1111/j.1365-294X.2005.02454.x

2. Talbot SL, Wyllie-Echeverria S, Ward DH, Rearick JR, Sage GK, Chesney B, et al. Genetic characterization of *Zostera asiatica* on the Pacific Coast of North America. Aquat Bot. 2006;85: 169–176. doi:10.1016/j.aquabot.2006.03.011

3. Reusch TBH, Stam WT, Olsen JL. A microsatellite-based estimation of clonal diversity and population subdivision in *Zostera marina*, a marine flowering plant. Mol Ecol. 2000;9: 127–140. doi:10.1046/j.1365-294X.2000.00839.x

4. Reusch TBH, Stam WT, Olsen JL. Microsatellite loci in eelgrass *Zostera marina* reveal marked polymorphism within and among populations. Mol Ecol. 1999;8: 317–321. doi:10.1046/j.1365-294X.1999.00531.x

5. Wyllie-Echeverria S, Talbot SL, Rearick JR. Genetic structure and diversity of *Zostera marina* (Eelgrass) in the San Juan Archipelago, Washington, USA. Estuaries and Coasts. 2010;33: 811–827. doi:10.1007/s12237-009-9243-z

6. Zhang Z, Schwartz S, Wagner L, Miller W. A greedy algorithm for aligning DNA sequences. J Comput Biol. 2000;7: 203–214.

7. Tanaka N, Kuo J, Omori Y, Nakaoka M, Aioi K. Phylogenetic relationships in the genera *Zostera* and *Heterozostera* (Zosteraceae) based on *matK* sequence data. J Plant Res. 2003;116: 273–279. doi:10.1007/s10265-003-0090-x

8. Kato Y, Aioi K, Omori Y, Takahata N, Satta Y. Phylogenetic analyses of *Zostera* species based on *rbcL* and *matK* nucleotide sequences: implications for the origin and diversification of seagrasses in Japanese waters. Genes Genet Syst. 2003;78: 329–342. doi:10.1266/ggs.78.329

9. Elven R. 230101-02 The *Zostera marina* aggregate *Z. angustifolia, Z. marina*. Panarctic flora (PAF) vascular plants [Internet]. 2007. Available: http://nhm2.uio.no/paf/flora#paf-230101-02

10. Becheler R, Diekmann O, Hily C, Moalic Y, Arnaud-Haond S. The concept of population in clonal organisms: mosaics of temporally colonized patches are forming highly diverse meadows of *Zostera marina* in Brittany. Mol Ecol. 2010;19: 2394–2407. doi:10.1111/j.1365-294X.2010.04649.x

11. Villesen P. FaBox: an online toolbox for FASTA sequences. Mol Ecol Notes. 2007;7: 965–968. doi:10.1111/j.1471-8286.2007.01821.x

12. Bandelt H-J, Forster P, Sykes BC, Richards MB. Mitochondrial portraits of human populations using median networks. Genetics. 1995;141: 743–753.

13. Park S. Microsatellite Toolkit for MS Excel 97 or 2000 (PC). Dublin: Stephen Park, Molecular Population Genetics Lab, Smurfit Institute of Genetics; 2000.

14. Paetkau D. An empirical exploration of data quality in DNA-based population inventories. Mol Ecol. 2003;12: 1375–1387. doi:10.1046/j.1365-294X.2003.01820.x

15. Arnaud-Haond S, Belkhir K. GENCLONE: a computer program to analyse genotypic data, test for clonality and describe spatial clonal organization. Mol Ecol Notes. 2007;7: 15–17. doi:10.1111/j.1471-8286.2006.01522.x

16. Dorken ME, Eckert CG. Severely reduced sexual reproduction in northern populations of a clonal plant, *Decodon verticillatus* (Lythraceae). J Ecol. 2001;89: 339–350. Available: http://www.jstor.org/stable/3072279

17. Waits LP, Luikart G, Taberlet P. Estimating the probability of identity among genotypes in natural populations: cautions and guidelines. Mol Ecol. 2001;10: 249–256. doi:10.1046/j.1365-294X.2001.01185.x

18. Valière N. GIMLET: a computer program for analysing genetic individual identification data. Mol Ecol Notes. 2002;2: 377–379. doi:10.1046/j.1471-8278

19. Lewis P, Zaykin D. Genetic data analysis: Computer program for the analysis of allelic data [Internet]. 2001. Available: http://hydrodictyon.eeb.uconn.edu/people/plewis/software.php

20. Peakall R, Smouse PE. GENALEX 6: Genetic analysis in Excel. Population genetic software for teaching and research. Mol Ecol Notes. 2006;6: 288–295. doi:10.1111/j.1471-8286.2005.01155.x

21. Petit RJ, el Mousadik A, Pons O. Identifying populations for conservation on the basis of genetic markers. Conserv Biol. 1998;12: 844–855.

22. El Mousadik A, Petit RJ. High level of genetic differentiation for allelic richness among populations of the argan tree [*Argania spinosa* (L.) Skeels] endemic to Morocco. Theor Appl Genet. 1996;92: 832–839.

23. Goudet J. FSTAT: a program to estimate and test gene diversities and fixation indices [Internet]. 2001. Available: http://www2.unil.ch/popgen/softwares/fstat.htm

24. Guo SW, Thompson EA. Performing the exact test of Hardy-Weinberg proportion for multiple alleles. Biometrics. 1992;48: 361–372.

25. Raymond M, Rousset F. Genepop (version 1.2): population-genetics software for exact tests and ecumenicism. J Hered. 1995;86: 248–249. Available: http://genepop.curtin.edu.au/

26. Rousset F. GENEPOP’007: a complete re-implementation of the GENEPOP software for Windows and Linux. Mol Ecol Resour. 2008;8: 103–106. doi:10.1111/j.1471-8286.2007.01931.x

27. Rice WR. Analyzing tables of statistical tests. Evolution (N Y). 1989;43: 223–225. doi:10.2307/2409177

28. Weir BS, Cockerham CC. Estimating F-statistics for the analysis of population structure. Evolution (N Y). 1984;38: 1358–1370.

29. Michalakis Y, Excoffier L. A generic estimation of population subdivision using distances between alleles with special reference for microsatellite loci. Genetics. 1996;142: 1061–1064.

30. Slatkin M. A measure of population subdivision based on microsatellite allele frequencies. Genetics. 1995;139: 457–462.

31. Goudet J, Raymond M, De Meeüs T, Rousset F. Testing differentiation in diploid populations. Genetics. 1996;144: 1933–1940. doi:10.1111/j.1471-8286.2007.01769.x

32. Excoffier L, Laval G, Schneider S. Arlequin (version 3.0): an integrated software package for population genetics data analysis. Evol Bioinform Online. 2005;1: 47–50. doi:10.1111/j.1755-0998.2010.02847.x

33. Ohta J, Kimura M. A model of mutation appropriate to estimate the number of electrophoretically detectable alleles in a finite population. Genet Res. 1973;22: 201–204.

34. Corander J, Waldmann P, Marttinen P, Sillanpää MJ. BAPS 2: enhanced possibilities for the analysis of genetic population structure. Bioinformatics. 2004;20: 2363–2369. doi:10.1093/bioinformatics/bth250

35. Pritchard JK, Stephens M, Donnelly P. Inference of population structure using multilocus genotype data. Genetics. 2000;155: 945–959. doi:10.1111/j.1471-8286.2007.01758.x

36. Jombart T, Devillard S, Balloux F. Discriminant analysis of principal components: a new method for the analysis of genetically structured populations. BMC Genet. BioMed Central Ltd; 2010;11: 94. doi:10.1186/1471-2156-11-94

37. Jombart T. adegenet: a R package for the multivariate analysis of genetic markers. Bioinformatics. 2008;24: 1403–1405. doi:10.1093/bioinformatics/btn129

38. Schneider S, Kueffer J, Roessli D, Excoffier L. Arlequin (version 1.1): An exploratory population genetics software environment. Geneva: Genetics and Biometry Laboratory, University of Geneva; 1997.

39. Saitou N, Nei M. The neighbor-joining method: a new method for reconstructing phylogenetic trees. Mol Biol Evol. 1987;4: 406–425.

40. Cavalli-Sforza LL, Edwards AWF. Phylogenetic analysis. Models and estimation procedures. Am J Hum Genet. 1967;19: 233–257. Available: http://www.pubmedcentral.nih.gov/articlerender.fcgi?artid=1706274&tool=pmcentrez&rendertype=abstract

41. Langella O. Populations 1.2.30: a population genetic software [Internet]. 2010. Available: http://bioinformatics.org/~tryphon/populations/

42. Page RDM. TreeView: an application to display phylogenetic trees on personal computers. Comput Appl Biosci CABIOS. 1996;12: 357–358. doi:10.1093/bioinformatics/12.4.357

43. Hutchison DW, Templeton AR. Correlation of pairwise genetic and geographic distance measures: inferring the relative influences of gene flow and drift on the distribution of genetic variability. Evolution (N Y). 1999;53: 1898–1914. doi:10.2307/2640449

44. Rousset F. Equilibrium values of measures of population subdivision for stepwise mutation processes. Genetics. 1996;142: 1357–1362.

45. Bohonak AJ. IBD (isolation by distance): a program for analyses of isolation by distance. J Hered. 2002;93: 153–154. doi:10.1093/jhered/93.2.153

46. Beerli P, Felsenstein J. Maximum-likelihood estimation of migration rates and effective population numbers in two populations using a coalescent approach. Genetics. 1999;152: 763–773. doi:10.1073/pnas.081068098

47. Beerli P, Felsenstein J. Maximum likelihood estimation of a migration matrix and effective population sizes in n subpopulations by using a coalescent approach. Proc Natl Acad Sci U S A. 2001;98: 4563–4568. doi:10.1073/pnas.081068098
