# Supplementary material for: The Structure of Genetic Diversity in Eelgrass (Zostera marina L.) along the North Pacific and Bering Sea Coasts of Alaska
Source: PLoS One. 2016 Apr 22;11(4):e0152701. doi: 10.1371/journal.pone.0152701 (PMC4841600; doi:10.1371/journal.pone.0152701)
Supplement: S1 Table — (DOCX) [file pone.0152701.s005.docx]

**S1 Table. Pairwise *θ_ST_* (below the diagonal) and ρ*_ST_* (above the diagonal) values among populations occupying the Bering Sea and North Pacific coasts of Alaska.**

|  | SL | KS | TOG | IZL | SCC | KIL | WB | UNGA | AKSI | PWS | NAK | YAB |
| --- | --- | --- | --- | --- | --- | --- | --- | --- | --- | --- | --- | --- |
| SL | - | **0.520** | **0.264** | **0.131** | **0.297** | **0.198** | **0.529** | 0.127 | 0.183 | **0.463** | **0.495** | **0.214** |
| KS | **0.371** | - | **0.107** | **0.563** | **0.795** | **0.109** | **0.449** | **0.725** | **0.618** | **0.090** | **0.205** | **0.437** |
| TOG | **0.324** | **0.100** | - | **0.336** | **0.515** | **-0.003** | **0.352** | **0.326** | 0.165 | **0.164** | **0.264** | **0.240** |
| IZL | **0.187** | **0.268** | **0.237** | - | **0.066** | **0.275** | **0.609** | -0.014 | 0.201 | **0.550** | **0.589** | **0.277** |
| SCC | **0.258** | **0.222** | **0.236** | **0.047** | - | **0.430** | **0.771** | 0.052 | **0.448** | **0.695** | **0.685** | **0.379** |
| KIL | **0.240** | **0.175** | **0.122** | **0.091** | **0.078** | - | **0.288** | 0.234 | 0.095 | **0.148** | **0.240** | **0.183** |
| WB | **0.515** | **0.592** | **0.582** | **0.477** | **0.483** | **0.473** | - | **0.666** | **0.469** | **0.162** | **0.103** | **0.322** |
| UNGA | **0.315** | **0.213** | **0.188** | **0.109** | **0.076** | **0.098** | **0.456** | - | 0.146 | **0.559** | **0.527** | 0.160 |
| AKSI | **0.407** | **0.290** | **0.234** | **0.283** | **0.232** | **0.172** | **0.386** | **0.119** | - | **0.367** | **0.330** | 0.021 |
| PWS | **0.309** | **0.231** | **0.223** | **0.201** | **0.160** | **0.115** | **0.368** | **0.122** | **0.113** | - | **0.069** | **0.318** |
| NAK | **0.417** | **0.290** | **0.279** | **0.297** | **0.257** | **0.192** | **0.412** | **0.147** | 0.043 | **0.119** | - | **0.308** |
| YAB | **0.354** | **0.368** | **0.382** | **0.308** | **0.247** | **0.257** | **0.369** | **0.218** | **0.192** | **0.159** | **0.217** | - |

EBS-LME populations = white background; GoA-LME populations = lighter shaded background. Values in bold are significant at *P <* 0.05 (Bonferroni correction applied; α = 0.005).
