# Supplementary material for: The Structure of Genetic Diversity in Eelgrass (Zostera marina L.) along the North Pacific and Bering Sea Coasts of Alaska
Source: PLoS One. 2016 Apr 22;11(4):e0152701. doi: 10.1371/journal.pone.0152701 (PMC4841600; doi:10.1371/journal.pone.0152701)
Supplement: S2 Table — (DOCX) [file pone.0152701.s006.docx]

**S2 Table. Analyses of molecular variance (AMOVA) for hypothesized groupings, based on fragment data from ten microsatellite loci.**

| **Model** | **Hypothesized groupings** | ***θ_F_*** | ***θ_f_*** | ***θ*_s_** | ***θ_p_*** | **% among groups** | ***P_θp_*** |
| --- | --- | --- | --- | --- | --- | --- | --- |
| A | [IZL,TOG,KS,SL,SCC] [KIL,WB,UNGA,AKSI,PWS,NAK] | **0.325** | **0.036** | **0.252** | 0.063 | 6.30 | 0.022 |
| B | [IZL,TOG,KS,SL,SCC] [KIL,WB,UNGA,AKSI] [PWS,NAK] | **0.314** | **0.036** | **0.260** | 0.040 | 4.02 | 0.139 |
| C | [IZL,TOG,KS,SL,SCC,KIL] [WB,UNGA,AKSI, PWS, NAK] | **0.351** | **0.036** | **0.227** | **0.128** | 12.79 | <0.001 |
| D | [IZL,TOG,KS,SL,SCC,KIL] [WB,UNGA,AKSI] [PWS,NAK] | **0.360** | **0.036** | **0.198** | **0.172** | 17.19 | 0.002 |
| E | [IZL,TOG,KS,SL] [SCC,KIL,WB,UNGA,AKSI] [PWS,NAK] | **0.310** | **0.036** | **0.268** | 0.022 | 2.22 | 0.205 |
| F | [IZL,TOG,KS,SL] [SCC,KIL,WB,UNGA,AKSI,PWS,NAK] | **0.318** | **0.036** | **0.262** | 0.041 | 4.15 | 0.096 |
| G | [IZL,KIL,SCC] [TOG,KS,SL] [WB,UNGA,AKSI,NAK,PWS] | **0.322** | **0.036** | **0.221** | 0.097 | 9.75 | 0.006 |
| H | [IZL,KIL,SCC] [TOG,KS,SL] [WB,UNGA,AKSI] [NAK,PWS] | **0.325** | 0.036 | **0.183** | **0.143** | 14.28 | <0.002 |

Fixation indices are shown, along with the percentage of the total variance that is explained by the hypothesized regional grouping and its significance. The first grouping (Model A) tests the hypothesis that genetic variation is partitioned between the Eastern Bering Sea and the GoA-LMEs. Values in bold for are significant at *P <* 0.005 (Bonferroni correction applied; α = 0.0004).
