# Supplementary material for: The Structure of Genetic Diversity in Eelgrass (Zostera marina L.) along the North Pacific and Bering Sea Coasts of Alaska
Source: PLoS One. 2016 Apr 22;11(4):e0152701. doi: 10.1371/journal.pone.0152701 (PMC4841600; doi:10.1371/journal.pone.0152701)
Supplement: S3 Table — (DOCX) [file pone.0152701.s007.docx]

**S3 Table. Pairwise estimates of directional gene flow (*N_e_m*) and Θ for each population, using 10 microsatellite loci, among EBS-LME populations.**

| *Providing Migrants* | | | | | | | |
| --- | --- | --- | --- | --- | --- | --- | --- |
| *Population* | | Θ | SCC | IZL | SL | KS | TOG |
| *Receiving Migrants* | SCC | 1.027 | __ | 2.906  (1.385-3.521) | 0.495  (0.340-0.700) | 0.382  (0.262-0.539) | 1.104  (0.820-1.469) |
|  | IZL | 0.969 | 3.361  (2.913 - 4.203) | __ | 0.838  (0.621-1.113) | 0.265  (0.166-0.395) | 1.264  (0.953-1.642) |
|  | SL | 0.971 | **1.227**  **(0.897-1.664)** | **3.555**  **(2.966-4.250)** | __ | 0.675  (0.504-0.889) | 1.609  (1.259-2.039) |
|  | KS | 0.991 | **1.121**  **(0.811-1.534)** | **1.4980**  **(1.160-1.923)** | 0.933  (0.701-1.226) | __ | **3.946**  **(3.298-4.710)** |
|  | TOG | 1.027 | 0.961  (0.677-1.344) | **2.703**  **(2.216-3.280)** | 1.428  (1.123-1.800) | 0.493  (0.353-0.671) | __ |

Populations listed vertically are receiving migrants; populations listed horizontally are providing migrants. For example, SCC is receiving 2.906 immigrants per generation from IZL, and the latter is receiving 3.361 immigrants from the former. Confidence intervals are listed in parentheses; instances of asymmetrical gene flow between population pairs are indicated in bold.
