# Supplementary material for: The Structure of Genetic Diversity in Eelgrass (Zostera marina L.) along the North Pacific and Bering Sea Coasts of Alaska
Source: PLoS One. 2016 Apr 22;11(4):e0152701. doi: 10.1371/journal.pone.0152701 (PMC4841600; doi:10.1371/journal.pone.0152701)
Supplement: S4 Table — (DOCX) [file pone.0152701.s008.docx]

**S4 Table. Pairwise estimates of directional gene flow (*N_e_m*) and Θ for each population, using 10 microsatellite loci, among GoA-LME populations.**

| *Providing Migrants* | | | | | | | | | |
| --- | --- | --- | --- | --- | --- | --- | --- | --- | --- |
| *Population* | | Θ | YAB | AKSI | KIL | WB | UNGA | NAK | PWS |
| *Receiving Migrants* | YAB | 1.002 | __ | 0.731  (0.480-1.094) | 0.952  (0.679-1.304) | 0.084  (0.037-0.164) | **0.720**  **(0.478-1.061)** | 0.750  (0.544-1.015) | **1.337**  **(0.975-1.799)** |
|  | AKSI | 0.924 | 0.860  (0.606-1.199) | __ | **2.432**  **(1.926-3.040)** | 0.647  (0.474-0.866) | 1.312  (0.943-1.805) | 1.117  (0.853-1.447) | **2.630**  **(2.050-3.330)** |
|  | KIL | 1.018 | 1.268  (0.942-1.685) | 1.051  (0.728-1.504) | __ | 0.183  (0.105-0.297) | 0.882  (0.603-1.267) | 0.610  (0.432-0.842) | **3.096**  **(2.469-3.853)** |
|  | WB | 0.983 | **0.910**  **(0.651-1.252)** | **1.512**  **(1.091-2.089)** | **2.051**  **(1.600-2.598)** | __ | 0.615  (0.398-0.926) | 0.931  (0.697-1.226) | **2.2679**  **(1.758-2.895)** |
|  | UNGA | 1.074 | 0.178  (0.089-0.320) | **2.513**  **(1.907-3.320)** | **3.303**  **(2.682-4.039)** | 0.323  (0.211-0.474) | __ | 0.6900  (0.497-0.938) | 0.728  (0.483-1.060) |
|  | NAK | 1.004 | 1.254  (0.931-1.668) | 0.975  (0.664-1.435) | **1.329**  **(0.988-1.757)** | 1.186  (0.929-1.499) | **1.521**  **(1.110-2.065)** | __ | 1.650  (1.235-2.173) |
|  | PWS | 1.038 | 0.614  (0.414-0.886) | 0.457  (0.275-0.733) | 1.051  (0.753-1.437) | 0.324  (0.213-0.475) | 0.424  (0.256-0.674) | 1.080  (0.822-1.404) | __ |

Populations listed vertically are receiving immigrants; populations listed horizontally are providing emigrants. Confidence intervals are listed in parentheses; instances of asymmetrical gene flow between population pairs are indicated in bold.
